# Supplementary material for: Development and validation of a multi-locus DNA metabarcoding method to identify endangered species in complex samples
Source: Gigascience. 2017 Aug 19;6(10):1–18. doi: 10.1093/gigascience/gix080 (PMC5632295; doi:10.1093/gigascience/gix080)
Supplement: GIGA-D-17-00107_Revision-2.pdf [file gix080_GIGA-D-17-00107_Revision-2.pdf]

Manuscript
Click here to download Manuscript A multi-locus DNA metabarcoding method_AJ_resubmission.docx

Development and validation of a multi-locus DNA metabarcoding method to identify endangered species in complex samples

Alfred J. Arulandhu [1, 2‡], Martijn Staats [1‡], Rico Hagelaar [1], Marleen M. Voorhuijzen [1], Theo W. Prins [1], Ingrid Scholtens [1], Adalberto Costessi [3], Danny Duijsings [3], François Rechenmann [4], Frédéric B. Gaspar [5], Maria Teresa Barreto Crespo [5], Arne Holst-Jensen [6], Matthew Birck [7], Malcolm Burns [8], Edward Haynes [9], Rupert Hochegger [10], Alexander Klingl [11], Lisa Lundberg [12], Chiara Natale [13], Hauke Niekamp [14], Elena Perri [15], Alessandra Barbante [15], Jean-Philippe Rosec [16], Ralf Seyfarth [17], Tereza Sovová [18], Christoff Van Moorleghem [19], Saskia van Ruth [1,2], Tamara Peelen [20] and Esther Kok [1 *]

Alfred J. Arulandhu [1] - RIKILT Wageningen University & Research, P.O. Box 230, 6700 AE Wageningen, The Netherlands – alfred.arulandhu@wur.nl

Alfred J. Arulandhu [2] – Food Quality and Design Group, Wageningen University and Research, P.O. Box 8129, 6700 EV Wageningen, The Netherlands – alfred.arulandhu@wur.nl

Martijn Staats [1] - RIKILT Wageningen University & Research, P.O. Box 230, 6700 AE Wageningen, The Netherlands – martijn.staats@wur.nl

Rico Hagelaar [1] - RIKILT Wageningen University & Research, P.O. Box 230, 6700 AE Wageningen, The Netherlands – rico.hagelaar@wur.nl

Marleen M. Voorhuijzen [1] - RIKILT Wageningen University & Research, P.O. Box 230, 6700 AE Wageningen, The Netherlands - marleen.voorhuijzen@wur.nl

Theo W. Prins [1] - RIKILT Wageningen University & Research, P.O. Box 230, 6700 AE Wageningen, The Netherlands - theo.prins@wur.nl

Ingrid M.J. Scholtens [1] - RIKILT Wageningen University & Research, P.O. Box 230, 6700 AE Wageningen, The Netherlands - ingrid.scholtens@wur.nl

Adalberto Costessi [3] - Baseclear B. V, Einsteinweg 5, 2333 CC Leiden, The Netherlands - Adalberto.Costessi@baseclear.nl

Danny Duijsings [3] - Baseclear B. V, Einsteinweg 5, 2333 CC Leiden, The Netherlands - Danny.Duijsings@baseclear.nl

François Rechenmann [4] - GenoStar Bioinformatics Solutions, 60 rue Lavoisier, 38330 Montbonnot Saint Martin, France - rechenmann@genostar.com

Frédéric B. Gaspar [5] – iBET, Instituto de Biologia Experimental e Tecnológica, Apartado 12, 2780-901 Oeiras, Portugal - fgaspar@ibet.pt

Maria Teresa Barreto Crespo [5] - iBET, Instituto de Biologia Experimental e Tecnológica, Apartado 12, 2780-901 Oeiras, Portugal - tcrespo@ibet.pt

Arne Holst-Jensen [6] - Norwegian Veterinary Institute, Ullevaalsveien 68, P.O.Box 750 Sentrum, 0106 Oslo, Norway - arne.holst-jensen@vetinst.no

Matthew Birck [7]- U.S. Customs and Border Protection Laboratory, 1100 Raymond Blvd Newark, NJ 07102 USA - MATTHEW.BIRCK@cbp.dhs.gov

Malcolm Burns [8] - LGC, Queens Road, Teddington, Middlesex, TW11 0LY, United kingdom - Malcolm.Burns@lgcgroup.com

Edward Haynes [9] – Fera, Sand Hutton, York, YO41 1LZ, United Kingdom - Edward.Haynes@fera.co.uk

Rupert Hochegger [10] - Austrian Agency for Health and Food Safety, Spargelfeldstrasse 191, 1220 Vienna, Austria - rupert.hochegger@ages.at

Alexander Klingl [11] – Generalzolldirektion, Direktion IX, Bildungs- und Wissenschaftszentrum der Bundesfinanzverwaltung, Dienstort Hamburg, Baumacker 3, D-22523 Hamburg, Germany - Alexander.Klingl@bwz.bund.de

Lisa Lundberg [12] - Livsmedelsverket, Att. Lisa Lundberg, Strandbodgatan 4, SE 75323 Uppsala, Sweden - lisa.lundberg@slv.se

Chiara Natale [13] - AGENZIA DELLE DOGANE E DEI MONOPOLI, Laboratori e servizi chimici – Laboratorio Chimico di Genova, 16126 Genova, Via Rubattino n.6, Italy - chiara.natale@agenziadogane.it

Hauke Niekamp [14] - Eurofins GeneScan GmbH, Engesserstrasse 4 79108 Freiburg, Germany - HaukeNiekamp@eurofins.de

Elena Perri [15] - CREA-SCS sede di Tavazzano - Laboratorio via Emilia, Km 307, 26838 Tavazzano, Italy - elena.perri@crea.gov.it

Alessandra Barbante [15] - CREA-SCS sede di Tavazzano - Laboratorio via Emilia, Km 307, 26838 Tavazzano, Italy - alessandra.barbante@crea.gov.it

Jean-Philippe Rosec [16] - Service Commun des Laboratoires, Laboratoire de Montpellier, Parc Euromédecine, 205 rue de la Croix Verte, 34196 Montpellier Cedex 5, France - Jean-Philippe.ROSEC@scl.finances.gouv.fr

Ralf Seyfarth [17] - Biolytix AG, Benkenstrasse 254, 4108 Witterswil, Switzerland - Ralf.seyfarth@biolytix.ch

Tereza Sovová [18] – Crop Research Institute, Department of Molecular Genetics, Drnovská 507, 161 06 Prague, Czech Republic - mail@terezasovova.cz

Christoff Van Moorleghem [19] - Laboratory of Customs & Excises, Blijde Inkomststraat 20, B-3000 Leuven, Belgium - christoff.vanmoorleghem@minfin.fed.be

Saskia van Ruth [1] - RIKILT Wageningen University & Research, P.O. Box 230, 6700 AE Wageningen, The Netherlands - saskia.vanruth@wur.nl

Saskia van Ruth [2] - Food Quality and Design Group, Wageningen University and Research, P.O. Box 8129, 6700 EV Wageningen, The Netherlands - saskia.vanruth@wur.nl

Tamara Peelen [20] - Dutch Customs Laboratory, Kingsfordweg 1, 1043 GN, Amsterdam, The Netherlands - t.peelen@belastingdienst.nl

Esther Kok [1*] - RIKILT Wageningen University & Research, P.O. Box 230, 6700 AE Wageningen, The Netherlands - esther.kok@wur.nl, ORCID: 0000-0003-1472-6710

‡ Alfred J. Arulandhu and Martijn Staats contributed equally to this work.

Corresponding author: Esther Kok
e-mail: esther.kok@wur.nl

Abstract (max. 250 words)

Background: DNA metabarcoding provides great potential for species identification in complex samples such as food supplements and traditional medicines. Such a method would aid CITES (the Convention on International Trade in Endangered Species of Wild Fauna and Flora) enforcement officers to combat wildlife crime by preventing illegal trade of endangered plant and animal species. The objective of this research was to develop a multi-locus DNA metabarcoding method for forensic wildlife species identification and to evaluate the applicability and reproducibility of this approach across different laboratories.

Results: A DNA metabarcoding method was developed that makes use of 12 DNA barcode markers that have demonstrated universal applicability across a wide range of plant and animal taxa, and that facilitate the identification of species in samples containing degraded DNA. The DNA metabarcoding method was developed based on Illumina MiSeq amplicon sequencing of well-defined experimental mixtures, for which a bioinformatics pipeline with user-friendly web interface was developed. The performance of the DNA metabarcoding method was assessed in an international validation trial by 16 laboratories, in which the method was found to be highly reproducible and sensitive enough to identify species present in a mixture at 1% dry weight content.

Conclusion: The advanced multi-locus DNA metabarcoding method assessed in this study provides reliable and detailed data on the composition of complex food products, including information on the presence of CITES-listed species. The method can provide improved resolution for species identification, while verifying species with multiple DNA barcodes contributes to an enhanced quality assurance.

Keywords: Endangered species, CITES, Traditional medicines, DNA metabarcoding, Customs agencies, COI, *matK*, *rbcL*, cyt *b*, mini-barcodes.

Background

The demand for endangered species as ingredients in traditional medicines (TMs) has become one of the major threats to the survival of a range of endangered species such as seahorse (*Hippocampus* sp.), agarwood (*Aquilaria* sp.), and Saiga antelope (*Saiga tatarica*) [1-3]. The Convention on the International Trade in Endangered Species of Wild Fauna and Flora (CITES) is one of the best supported conservation agreements to regulate trading of animal and plant species (www.cites.org) and thereby conserve biodiversity. Currently, ~35,000 species are classified and listed by CITES in three categories based on their extinction level (CITES Appendix I, II and III) by which the trade in endangered species is regulated. The success of CITES is dependent upon the ability of customs inspectors to recognize and identify components and ingredients derived from endangered species, for which a wide range of morphological, chromatographic and DNA-based identification techniques can be applied [4,5].

Recent studies have shown the potential of DNA metabarcoding for identifying endangered species in TMs and other wildlife forensic samples [4-7]. DNA metabarcoding is an approach that combines DNA barcoding with next-generation sequencing (NGS), which enables sensitive high-throughput multispecies identification on the basis of DNA extracted from complex samples [8]. DNA metabarcoding uses more or less universal PCR primers to mass-amplify informative DNA barcode sequences [9, 10]. Subsequently, the obtained DNA barcodes are sequenced and compared to a DNA sequence reference database from well-characterized species for taxonomic assignment [8, 10]. The main advantage of DNA metabarcoding over other identification techniques is that it permits the identification of all animal and plant species within samples that are composed of multiple ingredients, which would not be possible through morphological means and time-consuming with traditional DNA barcoding [4-6]. Furthermore, the use of mini-barcode markers in DNA metabarcoding facilitate the identification of species in highly processed samples containing heavily degraded DNA [5, 6]. Such a molecular approach could aid the Customs Authorities to identify materials derived from endangered species in a wide variety of complex samples, such as food supplements and TMs [11].

Before routine DNA metabarcoding can be applied, there are some key issues that need to be taken into account. First, complex products seized by Customs, such as TM products, may contain plant and animal components that are highly processed, and from which the isolation of good quality DNA is challenging. Second, the universal DNA barcodes employed may not result in amplification of the related barcode for each species contained in a complex sample, due to DNA degradation or the lack of PCR primer sequence universality. For plants, for example, different sets of DNA barcodes have been suggested for different fields of application (i.e.

general taxonomic identification of land plants, identification of medicinal plants, etc.), and none of them meet the true requirements of universal barcodes [12]. Also, whilst PCR primers can be designed to accommodate shorter DNA barcode regions for degraded DNA samples, such mini-barcodes contain less information and their primers are more restrictive, often making them unsuitable for universal species barcoding [4, 13]. The third challenge is the reference sequence database quality and integrity, which is particularly problematic for law enforcement issues, where high quality and reliability are essential. The current underrepresentation of DNA barcodes from species protected under CITES and closely related species critically hampers their identification. The fourth challenge is that a dedicated bioinformatics pipeline is necessary to process raw NGS data for accurate and sensitive identification of CITES-listed species [9]. Finally, studies using the DNA metabarcoding approach are scarce and none of these methods have been truly validated [9, 14]. Therefore, before implementing DNA metabarcoding by Customs and other enforcement agencies, the above-mentioned challenges need to be thoroughly assessed to ensure accurate taxonomic identifications.

The objective of this research was to develop a multi-locus DNA metabarcoding method for (endangered) species identification and to evaluate the applicability and reproducibility of this approach in an international interlaboratory study. The research was part of a larger programme on the development of advanced DNA-based methods from the DECATHLON project (www.decathlon-project.eu), within the European Union's Framework Programme 7. In the process of establishing the standard operating procedure (SOP) for multi-locus DNA metabarcoding, all important aspects of the procedure (i.e. DNA isolation procedure, DNA barcode marker, barcode primers, NGS strategy and bioinformatics) were evaluated. The challenges concerning the quality and integrity of the DNA reference database(s) are discussed. The first step was aimed at identifying an ideal DNA isolation method to extract DNA from complex mixtures consisting of both animal and plant tissues. Secondly, animal and plant DNA barcode markers and corresponding primer sets were identified from literature that allowed good resolution for identifying (endangered) species from a wide taxonomic range. Thirdly, a panel of universal plant and animal DNA barcodes was selected and a single optimal PCR protocol was identified for efficient amplification of a panel of DNA barcode markers. Finally, the suitability of the Illumina MiSeq NGS technology was evaluated, and a bioinformatics pipeline with a user-friendly web interface was established to allow stakeholders to perform the NGS data analysis without expert bioinformatics skills.

The DNA metabarcoding method was developed and tested based on data generated for 15 well-defined complex mixtures. The use of well-characterised mixtures allowed for optimising the bioinformatics procedure and subsequent robustness testing of multiple parameter settings and thresholds. The practical

performance and reproducibility of the DNA metabarcoding strategy was assessed in an international validation trial by 16 laboratories from 11 countries, on the basis of eight other newly composed complex mixtures and two seized TMs, which were suspected to contain ingredients derived from CITES species. In this study, the multi-locus DNA metabarcoding method is presented and it is assessed whether the method can improve the compositional analysis of complex and real-life samples by enabling the sensitive and reproducible identification of CITES-listed taxa by enforcement agencies and other laboratories.

Data description

To constitute well-defined complex mixtures, 46 reference specimens were commercially purchased from shops or were provided by the Dutch Custom Laboratory. In addition, two TMs that were suspected to comprise endangered species material were also obtained from Dutch Customs Laboratory. Each reference specimen was identified morphologically. Genomic DNA was extracted from 29 animal and 17 plant reference species for DNA barcoding. Standard cytochrome c oxidase I (COI) barcodes for all animal specimens were generated and individually sequenced using the Sanger method, and compared against the Barcode of Life Data Systems and NCBI database for taxonomic confirmation. For plant species, the DNA barcodes *rbcL* and *matK* were sequenced to confirm species identity. For a number of plant and animal species the generated barcode sequence information was deposited in the European Nucleotide Archive (ENA) under accession numbers LT009695 to LT009705, and LT718651 (Additional file 1; Table S1).

The complex mixtures for the pilot study and interlaboratory validation trial were prepared with 2 to 11 taxonomically well-characterised species present in relative concentrations (dry mass: dry mass) from 1% to 47%. For all experimental mixtures in the interlaboratory trial, internal control species were used to verify the efficiency of homogenization and to check for possible sample cross-contamination using species specific qPCR assays. DNA was isolated from the complex mixtures and the concentration and purity of extracted DNA was determined using spectrophotometer (NanoDrop 1000, Thermo Fisher Scientific Inc.). Subsequently, PCR amplifications using 12 DNA barcode primer sets were performed. The pooled and purified amplicons of each sample were sequenced using an Illumina MiSeq paired-end 300 technology, following the manufacturer's instructions (Illumina, Inc.). The NGS datasets were analysed using the CITESspeciesDetect pipeline. All raw NGS datasets from both analyses were deposited in ENA under accession numbers ERS1545972 to ERS1545988, ERS1546502 to ERS1546533, ERS1546540 to ERS1546619, ERS1546624 to ERS1546639, ERS1546742 to ERS1546757, ERS1546759 to ERS1546774, and study number PRJEB18620 (Additional file 3;

Table S1). A web interface was developed for the CITESspeciesDetect pipeline to allow stakeholders to perform the NGS data analysis of their own samples. The web interface can be globally accessed via the SURFsara high-performance computing and data infrastructure (http://decathlon-fp7.citespipe-wur.surf-hosted.nl:8080/).

Analyses

Establishing a laboratory procedure for multi-locus DNA barcode amplification

Based on the previous studies on DNA isolation for TMs [4, 15] and from the comparison between modified Qiagen DNeasy plant mini kit [16] and CTAB isolation [17] (unpublished results), we identified that the CTAB isolation method in general yields better DNA purity and provides better PCR amplification success. Therefore, the CTAB DNA isolation method was selected for successive experiments.

The DNA barcode markers included in this study were selected based on Staats et al. [9] supplemented with additional primers from literature [13] (Table 1). DNA barcode markers were selected based on the availability of universal primer sets and DNA sequence information in public repositories [9]. Important considerations in selecting suitable primer sets were that, preferably, they are used in DNA barcoding campaigns and studies, and as such have demonstrated universal applicability across a wide range of taxa. Furthermore, primer sets for both the amplification of full-length barcodes and their respective mini-barcodes (i.e. short barcode regions < 300 nt within existing ones) were selected when available. This was done to facilitate PCR amplification from a range of wildlife forensic samples containing relatively intact DNA (using full-length barcodes) and/or degraded DNA (mini-barcodes). Based on these criteria, PCR primer sets for the following animal DNA barcodes were selected: regions of the mitochondrial genes encoding 16S rRNA gene (16S), cytochrome c oxidase I (COI) and cytochrome *b* (cyt *b*). For plant species identification, primer sets for the following DNA barcodes were selected: regions of the plastidial genes encoding maturase K (*matK*), ribulose-1,5-bisphosphate carboxylase (*rbcL*), tRNA^{Leu} (UAA) intron sequence (*trnL* (UAA)), *psbA-trnH* intergenic spacer region (*psbA-trnH*), and the nuclear internal transcribed spacer 2 (ITS2) region (Table 1). The selected primers sets were modified to include the Illumina adapter sequence at the 5' end of the locus-specific sequence to facilitate efficient NGS library preparation. A gradient PCR experiment was performed to identify the optimal PCR annealing temperature. While the selected PCR primer sets had previously been published with their own annealing temperatures and conditions, the identification of a single optimal annealing temperature for all PCR primer sets would allow for increased efficiency of analysis. Initially, a thermal gradient of 49.0 °C to 55.0 °C was tested on the *Bos taurus* reference material with the primer sets for COI, 16S, mini-16S, and cyt *b*. The

amplification efficiency across the PCR primers sets was determined by comparing the intensity of the amplicons across the thermal gradient. An optimal annealing temperature of 49.5 ˚C was identified, but additional non-specific amplicons were observed with some primers (not shown). To reduce the amounts of non-specific amplification products, the PCR program was modified to increase the annealing temperature after five cycles from 49.5 ˚C to 54.0 ˚C [18], and tested on all 15 PCR primer sets (Table 1). It was observed that certain PCR primer combinations still produced non-specific products (for *psbA-trnH* gene) or less intense PCR products (for *rbcL* gene with primers rbcLa-F and rbcLajf634R, and *matK* gene with primers matK-390f and matK-1326r). Consequently, these PCR primer sets were excluded from subsequent experiments.

Next, the selected PCR thermocycling protocol was evaluated with the remaining 12 PCR primer sets on a panel of 29 animal and 17 plant species, representing a phylogenetically wide range of taxa (Mammalia, Actinopterygii, Malacostraca, Bivalvia, Aves, Reptilia, Amphibia, Insecta, Angiospermae, and Cycadopsida; Additional file 1; Table S2 and S3). The overall PCR amplification success rates varied across reference species and across DNA barcode markers (Additional file 1; Table S2). For instance, no PCR amplification was observed with cyt *b* for the CITES-listed species *Balaenoptera physalus*, whereas intense amplification was seen for the same species with 16S, COI, mini-16S and mini-COI (Additional file 1; Table S2). Overall, at least one DNA barcode marker could successfully be amplified for each of the 46 plant and animal species (Additional file 1; Table S2 and S3). For a number of plant and animal species the generated barcode sequence information was deposited in the European Nucleotide Archive (ENA) under accession numbers LT009695 to LT009705, and LT718651 (Additional file 1; Table S1).

Development and pre-validation of the CITESspeciesDetect bioinformatics pipeline

A dedicated bioinformatics pipeline, named CITESspeciesDetect, was developed for the purpose of rapid identification of CITES-listed species using Illumina paired-end sequencing technology. Illumina technology was selected because it produces NGS data with very low error rates, compared to other technologies [2, 19]. Furthermore, the Illumina MiSeq platform enables paired-end read lengths of up to 300 nt, allowing relatively long DNA barcode regions of up to ~550 nt to be assembled. Also, the multiplexing capabilities of Illumina technology are well developed, allowing for simultaneous sequencing of multiple samples in one run, thereby enabling more cost-efficient NGS. While NGS data analysis pipelines exist that allow processing of Illumina DNA metabarcoding datasets (e.g. CLOTU, QIIME, Mothur), the majority have been developed for specifically studying microbial communities using the 16S rRNA gene region. CITESspeciesDetect, developed in this study,

extends on the frequently-used software tools developed within the USEARCH [19] and BLAST+ packages [20], and additionally includes dedicated steps for quality filtering, sorting of reads per barcode, and CITES species identification (Figure 1). The CITESspeciesDetect is composed of five linked tools and data analysis passes through three phases: 1) pre-processing of paired-end Illumina data involving quality trimming and filtering of reads, followed by sorting by DNA barcode, 2) Operational Taxonomic Unit (OTU) clustering by barcode, and 3) taxonomy prediction and CITES identification.

It was found that with the current setup of the pipeline, reads generated for cyt *b* and mini-cyt *b* could not be separated based on the forward PCR primer, as the forward primers are identical. It was therefore decided to combine (pool) the overlapping reads of cyt *b* and mini-cyt *b* during pre-processing (primer selection) of reads to prevent reads from being double selected. This means that the results of cyt *b* and mini-cyt *b* are presented by the CITESspeciesDetect pipeline as cyt *b*. The same issue was found for COI barcode and mini-barcode markers, for which the results are presented as COI.

A parameter scan was performed in order to assess the effect of software settings on the ability to identify species. The evaluation allowed for the identification of important parameters and their effect on the sensitivity, specificity and robustness of the procedure. Changing the base quality score has a major impact on the number of reads per barcode (Additional file 1; Table S4). Increasing the strictness of the base quality score resulted in decreasing numbers of reads per barcode. Quality score values other than the default values (Q20 for 95% of bases) did not yield better identifications. When applying strict quality filtering settings (Q20 for 100% of bases, or Q30 for 99% of bases) the species *Pieris brassicae* and *Anguilla anguilla* could not be detected with cyt *b* and/or mini-COI, indicating these settings were too strict (Additional file 1; Table S5). This is likely due to the resulting overall low read numbers for cyt *b* and mini-COI when applying these strict quality filtering settings (Additional file 1; Table S4).

An OTU abundance threshold is generally applied to make DNA metabarcoding less sensitive to (potential) false-positive identifications. False-positives may occur e.g. as contaminants during pre-processing of samples (DNA extraction, PCR) or as cross-contamination during Illumina sequencing. Applying an OTU abundance threshold higher than zero generally results in loss of sensitivity. We have found, however, that applying an OTU abundance threshold of higher than zero may help in reducing noisy identifications and potential false-positive identifications (results not shown). It should be noted that applying filtering thresholds may always lead to false negative or false positive identifications. In this study, an OTU abundance threshold of

0.2% was set as default, however, the OTU abundance threshold may need re-evaluation for samples with expected very low species abundances (< 1% dry weight).

The effect of applying a minimum DNA barcode length revealed that allowing DNA barcodes of ≥ 10 nt did not lead to additional identification of species, compared with default settings (e.g. ≥ 200 nt). Increasing the minimal DNA barcode length to 250 nt, however, resulted in a failure to identify most plant species with mini-*rbcL* and *rbcL*. We implemented a minimum DNA barcode length of 200 nt, except for DNA barcodes with a basic length shorter than 200 nt, in which case the minimum expected DNA barcode length is set to 100 nt for ITS2, 140 nt for mini-*rbcL*, and 10 nt for the *trn*L (P6 loop) marker.

The results of the parameter scan resulted in specifying recommended parameter values (default setting) for analysing DNA metabarcoding datasets using the CITESspeciesDetect pipeline (see Methods section "Bioinformatics analysis"). An online version of the CITESspeciesDetect pipeline with a user-friendly web-interface was developed for skilled analysts with basic, but no expert level knowledge in bioinformatics and is made available via http://decathlon-fp7.citespipe-wur.surf-hosted.nl:8080/.

Pilot study to assess the performance of the DNA metabarcoding procedure using experimental mixtures

The DNA metabarcoding procedure was assessed in a pilot study, for which 15 complex mixtures (EM1 to EM15) were prepared containing from 2 to 10 taxonomically well-characterised species with DNA barcode reference sequences available in the NCBI reference database (Table 2). The experimental mixtures 10 and 11 (EM10 and EM11) were independently analysed twice to verify repeatability of the method (DNA isolation, barcode panel analysis and pooling). Only mixtures were used with well-characterised species (DNA Sanger barcoded and taxonomically verified) ingredients, at known dry weight concentrations, and with high quality DNA that would allow for an assessment of the performance of the DNA metabarcoding method under optimal conditions.

A total of 2.37 Gb of Illumina MiSeq sequencing data was generated for the 17 complex samples (15 complex mixtures along with the two replicates). On average, 464,648 raw forward and reverse Illumina reads were generated per sample, with minimum and maximum read numbers ranging between 273,104 (mixture EM4) and 723,130 (mixture EM10R; Table 3). During raw data pre-processing with the default settings of the CITESspeciesDetect pipeline, the reads were first quality filtered and overlapping paired-end Illumina reads were merged into pseudo-reads (Figure 1). The samples contained on average 269,099 quality controlled (QC) unmerged (forward and reverse) reads and merged pseudo-reads, collectively named (pseudo)reads. On average

88.27% (min = 77.38%, max = 96.26%) of raw reads passed the quality filtering and pre-processing steps, indicating that the overall quality of the Illumina data was high (not shown).

Next, the (pseudo)reads were assigned to DNA barcodes based on PCR primer sequences. On average, 96.44% (min = 88.78%, max = 98.21%) of QC pre-processed reads were assigned to DNA barcodes, indicating a high percentage of reads containing the locus-specific DNA barcode primers (Table 3). After this, the (pseudo)reads were clustered by 98% sequence similarity into OTUs. On average, 82.26% (min = 75.11%, max = 90.63%) of the DNA barcodes assigned reads were clustered into OTUs (Table 3). It was assumed that the small fraction of reads that was not assigned to OTUs contained non-informative (e.g. non-specific fragments, chimers) sequences that may have been generated during PCR amplification, and were filtered out during clustering.

For taxonomy prediction, OTUs were assigned to dataset sequences using BLAST when aligning with at least 98% sequence identity, a minimum of 90% query coverage, and an E-value of at least 0.001. Generally, the best match ("top hit") is used as best estimate of species identity. However, species identification using BLAST requires careful weighting of the evidence. To minimize erroneous taxonomic identifications a more conservative guideline was used that allowed a species to be assigned only when the best three matches identified the species. If the bit scores do not decrease after the top three hits, or if other species have identical bit scores, then identification was considered inconclusive. In such cases, OTUs were assigned to higher taxonomic levels (genus, family or order). All animal ingredients, except *Parapenaeopsis* sp. could be identified at the species-level with one or more DNA barcode marker using the default settings of the CITESspeciesDetect pipeline (Table 4 and 5). For plants, *Lactuca sativa* could be identified at the species-level using the *trn*L (P6 loop). All other plant taxa were identified at the genus or higher level (Table 4 and 5).

Putative contaminating species were observed in most of the experimental mixtures from multiple markers, detailed information about the identified cross-contained species in a sample and the related markers are specified in the Additional file 2; Table S1. Even with the default OTU abundance threshold in place, the species *L. sativa*, *B. taurus* and *Gallus gallus* were identified in mixtures that were not supposed to contain these species. To verify whether these putative contaminations occurred during DNA isolation or Illumina sequencing, qPCR assays for the specific detection of *B. taurus* and *G. gallus* were performed on selected DNA extracts. The high Cq values above 39 indicated the presence of these species, however, in low copy number, which suggests that for some experimental mixtures (EM8, EM9 and EM14) cross-contamination had occurred during sample preparation or DNA isolation, while for other experimental mixtures (EM15) cross-contamination may have

occurred during PCR, Illumina library preparation or sequencing. In addition to these contaminants, a species of *Brassica* was identified in experimental mixtures containing *P. brassica*. This result is most likely not a false-positive, because the caterpillars used for this study had been fed on cabbage.

The DNA metabarcoding method was found to be sensitive enough to identify most plant and animal taxa at 1% (dry mass: dry mass) in mixtures of both low (EM1, EM3 and EM5; Table 2) and relatively high complexity (EM6, EM8, EM11, EM12, and EM14; Table 2). The exception being *Parapenaeopsis* sp. (all mixtures), *A. anguilla* in EM6, and *Cycas revoluta* in EM8 and EM11. Careful inspection of the NGS data revealed that in nearly all cases OTUs related to *Parapenaeopsis* sp., *A. anguilla*, and *C. revoluta* were present, but that these sequences had been filtered out by the CITESspeciesDetect pipeline because their cluster sizes did not fulfil the 0.2% OTU abundance threshold. There appeared to be no trend as to the type and length of DNA barcode marker that had been filtered out by the CITESspeciesDetect pipeline. For instance, *Parapenaeopsis* sp. was detected below the OTU threshold with cyt *b*, mini-16S, COI, and 16S markers (not shown). Lowering the OTU abundance threshold, however, would lead to (more) false-positive identifications, and this was therefore not implemented.

The repeatability of the laboratory procedure (excluding NGS) was assessed by analysing the experimental mixtures 10 and 11 (EM10R and EM11R; Table 2), which was independently performed twice, i.e. DNA isolation and PCR barcode amplification, but NGS was performed on the same MiSeq flow cell as the other samples of the pilot study. From the comparison, it was observed that the percentage of QC reads was nearly twice as high in the replicate analyses (Table 3). Also, the percentage of QC reads assigned to DNA barcodes varied among replicate analyses (Figure 2). Most notable were the observed differences among replicate analyses in the percentage reads assigned to *matK* and the *trnL* (P6 loop). For example, the percentage of QC reads assigned to *matK* were 6.11% (14081 reads) and 0.02% (97 reads) in EM10 and EM10R respectively (Figure 2). The low number of reads assigned to *matK* limited its use for taxonomy identification in EM10R (Table 4). The multi-locus approach, however, allowed for the repeatable identification of taxa in EM10 and EM11, though not in all cases with all DNA barcode markers (Table 4 and 5).

Based on the results obtained from the pilot study, precautions were taken when grinding the freeze-dried materials and subsequent mixing to avoid cross-contamination during the laboratory handling of samples, which were used to improve the SOP for the interlaboratory trial (see protocols in [21])(dx.doi.org/10.17504/protocols.io.ixbcfin). Also, control species were added to experimental mixtures that

were prepared for the inter-laboratory trial to allow better confirmation of sample homogeneity and to verify that no cross-contamination had occurred during sample preparation.

Assessment of interlaboratory reproducibility of the DNA metabarcoding procedure

Altogether 16 laboratories from 11 countries (all experienced, well-equipped and proficient in advanced molecular analysis work), including two of the method developers, participated in the inter-laboratory trial (Table 6). The laboratories received ten anonymously labelled samples, each consisting of 250 mg powdered material. Two of the samples, labelled S3 and S8, were authentic TM products seized by the Dutch Customs Laboratory while the other eight samples were well-characterized mixtures of specimens from carefully identified taxa in relative dry weight concentrations from 1% to 47% (Table 7). In all experimental mixtures, 1% of *Zea mays* was added as quality control for homogeneity, which was confirmed with maize-specific *hmg* (high-mobility group gene) qPCR [16]. Also, tests performed with species-specific qPCR assays indicated that cross-contamination did not occur during sample preparation (Additional file 1; Table S6). The qPCR assay for the detection of *Brassica napus*, however, also gave a positive signal for other *Brassica* sp. in the mixtures.

Together with the sample materials, reagents for DNA extraction, and the complete set of barcode primers, the participants received an obligatory SOP. Any deviations from the SOP had to be reported. The participants were instructed to extract DNA, perform PCR using the barcode primers, purify the amplified DNA by removal of unincorporated primers and primer dimers, and assess the quality and quantity of the amplification products by gel electrophoresis and UV-spectrophotometry. The purified PCR products were then collected by the coordinator of the trial (RIKILT Wageningen University & Research, the Netherlands) and shipped to a sequencing laboratory (BaseClear, the Netherlands) for Illumina sequencing using MiSeq PE300 technology. The sequencing laboratory performed Index PCR and Illumina library preparation prior to MiSeq sequencing as specified in the Illumina 16S metagenomics sequencing library preparation guide. The altogether 160 PCR samples were sequenced using two Illumina flow cells with MiSeq reagent kit v3.

The interlaboratory trial should ideally have included the use of the online version of the pipeline, but unfortunately this was not possible due to shortage of time. Therefore, a single (developer) laboratory performed these bioinformatics analyses. The 160 individual samples contained on average 269,057 raw reads, and more than 150,000 reads per sample in 95% of the samples (Additional file 1; Table S7). One sample contained less than 100,000 reads (51,750), which was considered more than sufficient for reliable species identification. After pre-processing, the samples contained on average 142,938 (pseudo)reads. On average 94.66% of the reads (min

= 88.12%, max = 98.02%) passed the quality filtering indicating that the overall quality of the sequence data was consistently high across the 160 datasets.

OTU-clustering at 98% sequence similarity on average assigned 78.14% of the pre-processed and DNA barcode assigned reads into OTUs (Additional file 1; Table S7). Only two samples, both from the same laboratory, had a slightly lower percentage of the (pseudo-)reads assigned to OTUs (66.02% and 66.05%). This indicates that the pipeline correctly removed PCR artefacts in the clustering phase.

For taxonomy prediction, an OTU would be assigned to a database hit if they aligned with ≥ 98% sequence identity and ≥ 90% query coverage, and yielded an expect value (E-value) of at least 0.001. The BLAST output of the NGS data was interpreted by participants according to the guidelines in the SOP. Variation was observed among laboratories in interpreting the BLAST output: some laboratories consistently scored the top hits, irrespective of bitscore, while other labs selected all hits belonging to the top three bitscores, or interpreted only the first OTU of each DNA barcode, leading to large differences in identified taxa. Because of these inconsistencies, the BLAST results were re-interpreted by RIKILT Wageningen University & Research following the established guideline as mentioned in the SOP. These re-interpreted data are the data referred to in the following sections.

With one exception, all taxa mixed in at ≥ 1% (dry mass: dry mass) were reproducibly identified by at least 13 (81%) laboratories (Table 7). *Beta vulgaris* in sample S6 could only be identified by 4 out of 16 (25%) laboratories. *Beta vulgaris* specific sequences were present in all remaining datasets, but at very low read counts. So these clusters did not fulfil the 0.2% OTU abundance threshold (Additional file 2; Table S2) . In order to provide insight into what alternative setting of the CITESspeciesDetect pipeline may have been better suited for identifying *Beta vulgaris*, three data sets with relatively low (S6 – laboratory 13), medium (S6 – laboratory 14) and high (S6 – laboratory 6) data volumes were reanalysed using a range of different settings for the OTU minimum cluster size and OTU abundance threshold (Additional file 2: Table S3-S5). Setting the OTU minimum cluster size to 2, 4, or 6 has no effect on taxon identification, and *Beta vulgaris* is not identified at the species or higher taxonomic level in the data sets of laboratories 6 and 13. Setting the OTU abundance threshold to zero allows identifying *Beta vulgaris* in all three samples, but at the expense of many false positive identifications. Applying an OTU abundance threshold of 0.1% (default is 0.2%) allows identifying *Beta vulgaris* at the species or genus level irrespective of any differences in data volume between the three samples.

All six animal species could be identified to species level with at least one barcode marker (COI), while only four of the 12 plant species (*Brassica oleracea*, *Carica papaya*, *Gossypium hirsutum*, and *L. sativa*) could

be identified to species level (Additional file 2; Table S6). All other plant species were identified at the genus or higher level. For plants, no single barcode marker was best, and the most reliable data were obtained by combining the plant barcodes.

Three taxa that were misidentified or not intentionally included in the mixtures were reproducibly identified across all laboratories. *Acipenser schrenckii* co-occurred in all samples containing *Huso dauricus*. We have confirmed with DNA metabarcoding that the caviar used for preparing the experimental mixtures contains both *H. dauricus* and *A. schrenckii* (results not shown). Furthermore, *Brassica rapa* was identified by ITS2 in sample S4 by all 16 (100%) laboratories, instead of *Brassica napus*. We confirmed by Sanger sequencing *rbcL* and *matK* that our reference specimen is indeed *Brassica napus*, but that its ITS2 sequence is identical to *Brassica rapa* (LT718651). Finally, a taxon of the plant family Phellinaceae was reproducibly identified (by all laboratories) using the mini-*rbcL* marker in all samples containing *L. sativa* (S6, S7, S9, S10). Species of the family Phellinaceae and *L. sativa* both belong to the order Asterales. The evidence for Phellinaceae was not strong, i.e. the family-level identification was based on a single NCBI reference sequence only (GenBank: X69748). We therefore suspect a misidentification during the interpretation of the BLAST results.

Taxa that were identified to be the result of possible contaminations were scarcely observed, i.e. these were found in isolated cases and could possibly be explained by cross-sample contamination that may have occurred during any step of sample processing (DNA isolation, PCR, NGS library preparation or NGS). For example, a contamination with *Gossypium* sp. was observed using *trn*L (P6 loop) in sample S1 of one of the participating labs. A total of 6 of such suspected cases of incidental cross-contaminations were observed (not shown).

For the authentic TMs S3 and S8, it was observed that only few labelled ingredients could reproducibly be identified (Table 8 and 9). For sample S3 (Ma pak leung sea-dog), only the listed ingredients *Cuscuta* sp. (Chinese dodder seed), and *Astragalus danicus* (Astragalus root) could be identified. For sample S8 (Cobra performance enhancer), only the listed ingredients *Epimedium* sp. (Horny goat weed; Berberidaceae), *Panax ginseng* (Korean ginseng; Araliaceae), and species of the plant families Arecaceae (*Serenoa repens*) and Rubiaceae (*Pausinystalia johimbe*) could be identified. While most declared taxa were not identified, many non-declared taxa were identified. For sample S3, the animal species *B. taurus*, and the plants *Cullen* sp. (Fabaceae), *Melilotus officinalis* (Fabaceae), *Medicago* sp. (Fabaceae), *Bupleurum* sp. (Apiaceae), and *Rubus* sp. (Rosaceae) were identified by at least 14 (88%) laboratories (Table 8). Furthermore, the fungi *Aspergillus fumigatus* (Aspergillaceae) and *Fusarium* sp. (Nectriaceae) were reproducibly identified, of which the former is also a

known human pathogenic fungus. For sample S8, the animal species *B. taurus* and *Homo sapiens*, the plant species *Sanguisorba officinalis* and *Eleutherococcus sessiliflorus*, and members of the plant genera *Croton* and *Erythroxylum*, and families Meliaceae and Asteraceae, were reproducibly identified (Table 9).

Discussion

In this study, a DNA metabarcoding method was developed using a multi-locus panel of DNA barcodes for the identification of CITES protected species in highly complex products such as TMs. As a first step, a CTAB DNA isolation method was selected for efficiently extracting high quality DNA from pure plant and animal reference materials as well as from complex mixtures. DNA isolation can be very difficult to standardise and optimise because of the complexity and diversity of wild life forensic samples, and a more systematic comparison of different DNA extraction methods is required. Secondly, a single PCR protocol, suitable for all the barcodes included, i.e. multiple universal plant and animal barcode and mini-barcode markers, was identified. This facilitated the design of a multi-locus panel of DNA barcodes. Furthermore, the developed DNA metabarcoding method includes a dedicated bioinformatics workflow, named CITESspeciesDetect, that was specifically developed for the analysis of Illumina paired-end reads. The developed pipeline requires skilled experts in bioinformatics, and applies scripts for command-line processing. NGS data analysis pipelines may provide a lot of flexibility to the user, as modifications are easily implemented by expert users. The design of the pipeline prevented cyt *b* and COI full-length barcodes to be separated from their corresponding mini-barcodes, as they have identical forward primers. Since, the 300 PE reads can read through the cyt *b* and COI mini-barcodes, and therefore contain both 5'primer and 3'primer information, separation should be feasible.

To simplify the inter-laboratory validation of the pipeline, a user-friendly and intuitive web-interface with associated "Help" functions and "FAQs" was developed for the CITESspeciesDetect pipeline. The web interface was, however, not available in the course of the interlaboratory trial. Therefore, the sequence data generated in the interlaboratory study could not be analysed by the individual laboratories using the CITESspeciesDetect pipeline. A single (developer) laboratory therefore performed these analyses. Upon the availability of the online web-interface, individual participants were later given the opportunity to reanalyse their DNA metabarcoding data. Observations made in this part demonstrated concordance of results with those obtained by the developing laboratory, reinforcing the perception of CITESspeciesDetect as a user-friendly and reliable pipeline that may readily be used by enforcement agencies and other laboratories.

The performance of the DNA metabarcoding method was assessed in an interlaboratory trial in which the method was found to be highly reproducible across laboratories, and sensitive enough to identify species

present at 1% dry weight content in experimental samples containing up to 11 different species as ingredients. However, not all laboratories could identify all specified ingredients (species) in the analysed experimental samples. From the current study, we demonstrate that diverse animal taxa could be identified at the species level, which highlights the object of the method to target a wide range of animal species. COI (full-length COI and mini-COI) was found to be the most effective DNA barcode marker for animal species identification. This is not surprising considering that COI is the standard barcode for almost all animal groups [22]. Nearly all animal species identifications were supported by multiple DNA barcodes, thereby giving strong confidence to the correctness of the animal species identifications. In contrast, plants could mainly be identified at the family level, and no single DNA barcode marker was found to provide best resolution for identifying plant taxa. Ideally, adequate plant species discrimination would require the combined use of multiple DNA barcode markers, e.g. *rbcL* + *matK* [23], but this is technically not possible due to the nature of the target samples (heavily processed) and with the current Illumina Miseq technology. For the identification of plant taxa listed by CITES, the use of DNA barcodes with relatively modest discriminatory power at the genus or higher taxonomic level can still be useful, as it is often an entire plant genus or family that is listed by CITES, rather than individual plant species. This was the case for e.g. Orchidaceae and Cactaceae in this study. Yet, for some plant species (e.g. *Aloe variegata*) the resolution provided by the used plant DNA barcodes may still be too low for unambiguous CITES identification. It is important to note that the maximum achievable Illumina NGS read length limits the taxonomic resolution of DNA barcodes that are longer than ~550 nt. This particularly limited the discriminatory power of the full-length plant barcodes *matK* and *rbcL*. The DNA metabarcoding method may therefore benefit from (currently unavailable) Illumina read lengths longer than 300 nt, or other long-read sequencing technologies. Alternatively, full-length barcodes may be resolved using an advanced bioinformatics strategy (SOAPBarcode) to assemble Illumina shotgun sequences of PCR amplicons [24]. Single barcodes in several cases failed to amplify or provide resolution. The latter is likely to be caused mainly by database incompleteness, lack of genetic variability within some loci/target sequences, and sample composition. However, combining multiple barcodes into a multi-locus metabarcoding method mitigated the problems observed for individual barcodes. A high degree of confidence in the taxonomic assignments based on the combined barcodes were therefore observed, providing for enhanced quality assurance compared to the use of single barcodes.

While the use of well-characterised experimental mixtures allowed for an assessment of the performance of the DNA metabarcoding method under ideal conditions, the amplifiable DNA content of real-life samples encountered in routine diagnostic work are often of an unpredictable and variable quality. An analysis of

two authentic TM products seized by the Dutch Customs Laboratory demonstrated that only few ingredients listed on the labels could be reproducibly identified. This does not mean that the undetected species were not used as ingredients. Ingredients may have been processed in such a way that the DNA is either degraded or effectively removed. This is e.g. the case with refined oils or cooked ingredients [25]. A PCR-free targeted DNA capturing approach coupled with shotgun sequencing was recently proposed for biodiversity assessments which may potentially also be suitable for enhancing species identification in difficult wildlife forensic samples [24, 26]. The quality of the sequence reference database also strongly affects the ability to correctly identify species. Without correct references that also exhibit the necessary intraspecific variation, it is not possible to match and discriminate sequence reads correctly. It is well-known that accurate DNA barcoding depends on the use of a reference database that provides good taxonomic coverage [5, 9]. The current underrepresentation of DNA barcodes from species protected by CITES and closely related species critically hampers their identification. We estimate that only 18.8% of species on the CITES list contain one or more DNA barcodes (COI for animals, and *matK* or *rbcL* for plants). This will improve as DNA barcoding campaigns continue, in particular through initiatives such as the Barcode of Wildlife Project (BWP; www.barcodeofwildlife.org). Only by expansion of the sequence reference database of endangered and illegally-traded species can DNA barcoding provide the definitiveness required in a court of law.

A noteworthy observation was that most species that were reproducibly identified did not appear on the ingredients lists on the labels of the analysed TMs. This is possibly due to mislabelling. If the identifications are correct this also indicates that consumption may pose health risks. These findings corroborate earlier reports that DNA metabarcoding may provide valuable information about the quality and safety of TMs [5, 6].

Potential implications

Overall, our findings demonstrate that the multi-locus DNA metabarcoding method assessed in this study can provide reliable and detailed data on the composition of highly complex food products and supplements. This study highlights the necessity of a multi-locus DNA metabarcoding strategy for species identification in complex samples, since the use of multiple barcode markers can enable an increased resolution and quality assurance, even in heavily processed samples. The developed robust bioinformatics pipeline for Illumina data analysis with user-friendly web interface allows the method to be directly applied in various fields such as: a) food mislabelling and fraud in the food industry [27], b) environmental monitoring of species [28], and c) wildlife forensics [29]. Furthermore, the pipeline can be readily used to analyse different types of Illumina paired-end

datasets, even the future Illumina datasets (read length > 300 nt). Additionally, the web interface provides an opportunity for the global audience with limited expertise in bioinformatics, to analyse their own data. It also provides the liberty to select different primer sets and customise the settings for the selected purposes. As a result, the range of potential applications of the method to identify plant and animal species is diverse, the pipeline is versatile and adjustable to the user's needs, thus providing a powerful tool for research as well as enforcement purposes.

Methods

Reference materials and preparation of experimental mixtures

All reference specimens were obtained from a local shop in the Netherlands or provided by the Dutch Customs Laboratory (Additional file 1; Table S2 and Table S3). The reference specimens were taxonomically characterised to the finest possible taxonomic level. For each species, it was checked whether reference sequences were present in NCBI GenBank. For taxonomic confirmation, standard COI barcodes for all animal specimens were generated and individually Sanger sequenced, and compared against the NCBI and BOLD nucleotide database. For plant species, the DNA barcodes *rbcL* and *matK* were Sanger sequenced to confirm species identity. For a number of plant and animal species the generated barcode sequence information was deposited in the European Nucleotide Archive (ENA) under accession numbers LT009695 to LT009705, and LT718651 (Additional file 1; Table S1).

For the initial pilot study, in which the SOP for the DNA metabarcoding approach was established and tested, 15 well-defined complex mixtures were artificially prepared (Table 2). These experimental mixtures were prepared with 2 to 10 taxonomically well-characterised species (Table 2). The ingredients were mixed based on dry weight ratio, for which individual materials were freeze-dried for 78 hours. The lyophilized ingredients were ground using an autoclaved mortar and pestle or blender in a cleaned fume hood, and subsequently stored at -20 °C ˚C. The individual ingredients of each complex mixture were weighted and mixed thoroughly using a tumbler (Heidolph Reax 2) for 20 hours and stored at -20 ˚C until further use.

For the interlaboratory validation trial, in which the applicability and reproducibility of the DNA metabarcoding method was assessed, eight additional well-characterised mixtures were artificially prepared using the above procedure. These complex mixtures were prepared with 8 to 11 taxonomically well-characterised species present at dry weight concentrations from 1% to 47% (Table 7). These complex mixtures were prepared in such a way that the efficiency of homogenization and possibility of sample cross-contamination

could be verified using species-specific qPCR assays. In all samples, 1% of *Zea mays* was added as quality control for homogeneity. The presence of *Z. mays* was checked after sample mixing using maize-specific *hmg* qPCR along with a positive and negative control. A unique species was added at 1% dry weight to each mixture (S1-*Glycine max,* S2-*Gossypium* sp.,S4-*Brassica napus,* S5-*Triticum aestivum,* S6-*Beta vulgaris,* S7-*Meleagris gallopavo,* S9-*Carica papaya,* S10-*Solanum lycopersicum*) (Table 7). Species-specific qPCR was performed in duplex (together with positive and negative controls) in all samples, to check for possible cross-contamination between samples after sample preparation. Information about the qPCR primers and probes, and qPCR procedure can be found in the Additional file 1; Table S8-S10. In addition to the eight experimental mixtures, two TMs were included that were obtained from the Dutch Customs Laboratory: a) Ma pak leung sea-dog hard capsules (MA PAK LEUNG CO, LTD, Hong Kong), was labelled to contain among others rhizoma Cibotii (*Cibotium barometz,* CITES appendix II), and Herba Cistanches (*Cistanche* sp., CITES appendix II) and b) Cobra performance enhancer hard capsules (Gold caps, USA), was labelled to contain among others Siberian ginseng (*Eleutherococcus senticosus*) and Korean ginseng (*Panax ginseng*). In both TMs, the medicine powder was encapsulated in a hard-capsule shell. All capsules were opened and the powder inside the capsules were stored in air-sealed and sterilized containers. The powdered medicines were thoroughly mixed using tumbler (Heidolph Reax 2) for 20 hours and stored at -20 ˚C until further use.

DNA isolation method

A cetyltrimethylammonium bromide (CTAB) extraction method [17] was assessed for its ability to efficiently extract DNA from a range of plant and animal materials (SOP). In brief, the CTAB method consists of an initial step to separate polysaccharides and organic soluble molecules using a CTAB extraction buffer (1X CTAB, 1.4M NaCl, 0.1 M Tris-HCl [pH 8.0], and 20mM NA_2EDTA) and chloroform. Next, the DNA was precipitated with 96% ethanol, purified with 70% ethanol, and the obtained DNA was stored at 4 ˚C until further use. DNA was extracted from 100 mg reference materials (plant and animal), artificially made complex mixtures, and real-life samples (TMs) along with an extraction control. The concentration and purity ($OD_{260/280}$ and $OD_{260/230}$ ratios) of the obtained DNA was determined by spectrophotometer (NanoDrop 1000 instrument, Thermo Fisher Scientific Inc.). The $OD_{260/280}$ ratios between 1.7 and 2.0 were considered to indicate purity of the obtained DNA. In case the extraction control contained DNA, the DNA isolation procedure was repeated.

Barcode markers

Candidate universal DNA barcode and mini-barcode markers and primer sets were identified using the information provided in Staats et al. (2016) [9], supplemented with additional primer sets from literature (Table 1). The PCR primer sets were modified to have an additional Illumina tail sequence at 5' end of the primers (Table 1).

PCR

A gradient PCR was performed with all PCR primer combinations using 10 ng of DNA. The tested PCR conditions programme were according to the following protocol: 95 ˚C for 15 min, five cycles of 94 ˚C for 30 s, annealing range (49-55 ˚C) for 40 s, and 72 ˚C for 60 s, followed by 35 cycles of 94 ˚C for 30 s, 54 ˚C for 40 s, and 72 ˚C for 60 s, with a final extension at 72 ˚C for 10 min. The total volume of the PCR mixture was 25 µl, which included 12.5 µl of HotStarTaq Master Mix (Qiagen), 0.5 µl of 10 µM each sense and antisense primer, 7 µl of RNase-free water (Qiagen) and 5 µl of 10 ng/µl of represented species DNA. PCR was performed in the CFX96 thermal cycler (Bio-Rad) and the amplified products from all the analysed reference specimens, artificially made complex mixtures, and real-life samples (TMs) together with the positive and negative control reactions were visualised on 1% agarose gels. If amplification was observed in the negative control, the PCR analysis was repeated. Prior to NGS library preparation, 8 µl of PCR product of each target (12 in total) per sample was pooled and mixed. Next, the pooled PCR products were purified using the QIAquick PCR purification kit (Qiagen) according to manufacturer's protocol, and the purified amplicons were visualized on 1% agarose gels for all the artificially made complex mixtures, and real-life samples (TMs).

Next Generation Sequencing

The pooled and purified PCR amplicons were sequenced using Illumina MiSeq paired-end 300 technology. Prior to MiSeq sequencing, Index PCR and Illumina library preparation were performed as specified in the Illumina 16S metagenomics sequencing library preparation guide [30]. All the DNA barcode amplicons of each sample were treated as one sample during library preparation i.e. all DNA barcode amplicons of each sample were tagged with the addition of the same, unique identifier, or index sequence, during library preparation. The Index PCR was performed to add dual indices (multiplex identifiers) and Illumina sequencing adapters using the Nextera XT Index Kit (Illumina, FC-131-1001). The prepared Illumina libraries from each sample were

quantified using the Quant-iT dsDNA broad range assay (Life Technologies). Furthermore, the normalised library pools were prepared and their concentration was quantified using KAPA library quantification kit (KAPA Biosystems) and pooled prior to MiSeq sequencing using MiSeq reagent kit v3.

Bioinformatics analysis

The raw demultiplexed Illumina reads with Illumina 1.8+ encoding were processed using a bioinformatics pipeline, called CITESspeciesDetect. The CITESspeciesDetect is composed of five linked tools with data analysis passing through three phases: 1) pre-processing of paired-end Illumina data involving quality trimming and filtering of reads, followed by sorting by DNA barcode, 2) OTU clustering by barcode, and 3) taxonomy prediction and CITES identification (Figure 1).

During preprocessing of reads, the 5' and 3' Illumina adapter sequences are trimmed using Cutadapt v1.9.1 (cutadapt, RRID:SCR_011841) [31] using the respective substrings TGTGTATAAGAGACAG and CTGTCTCTTATACACA. After Illumina adapter trimming, reads ≤ 10 bp are removed using Cutadapt. Then, the forward and reverse reads are merged to convert a pair into a single pseudoread containing one sequence and one set of quality score using USEARCH v8.1.1861 [19].

Next, the merged pseudo-reads, unmerged forward reads and unmerged reverse reads are processed separately during quality filtering using a sliding window method implemented in PRINSEQ (PRINSEQ, RRID:SCR_005454) [32]. During this procedure, low quality bases with Phred scores lower than 20 are trimmed from 3'-end using a window size of 15 nt and a step size of 5 nt. After PRINSEQ, reads with a minimum of 95% per base quality ≥ 20 are kept, while the remaining reads are removed using FASTX_Toolkit v0.0.14 [32]. Then, reads are successively selected, trimmed and sorted per DNA barcode marker using Cutadapt [31]. The following steps are followed for each DNA barcode marker separately during this procedure. First, reads containing an anchored 5' forward primer or anchored 5' reverse primer (or their reverse complement) are selected with a maximum error tolerance of 0.2 (=20%) and with the overlap parameter specified to 6 to ensure specific selection of reads. Also, reads ≤ 10 nt are removed. The anchored 5' primer sequences are subsequently trimmed. Second, primer sequences that are present at the 3' end of the selected reads are also removed. For each DNA barcode, the primer-selected and unmerged reverse reads are reverse complemented and combined with primer-selected merged and unmerged forward reads.

The following procedure is used to cluster the quality trimmed reads of each DNA barcode into OTUs using the UPARSE pipeline implemented in USEARCH [19] with the following modifications: reads are

dereplicated using the derep_prefix command. Also, singleton reads and reads with minimum cluster size smaller than 4 are discarded. Representative OTUs are generated using an OTU radius of 2 (98% identity threshold) and 0.2% OTU abundance threshold with minimum barcode length per primer set. Filtering of chimeric reads is performed using the default settings of the UPARSE-REF algorithm implemented in the cluster_otus command of USEARCH.

To assign OTUs to taxonomy, standalone BLASTn megablast searches (BLASTN, RRID:SCR_001598) [20] of representative OTUs are performed on the National Centre for Biotechnology Information (NCBI) GenBank nucleotide database using an Expectation value (E-value) threshold of 0.001 and a maximum of 20 aligned sequences. OTUs are assigned to the database sequence to which they align, based on bit score, and having at least 98% sequence identity and minimum of 90% query coverage. To identify putative CITES-listed taxa, the taxon ID first was matched against the NCBI taxonomy database using Entrez Direct (edirect) functions (available at ftp://ftp.ncbi.nlm.nih.gov/entrez/entrezdirect/) to retrieve scientific name (species, genus, family, order and synonym name). The scientific, synonym and/or family names are then matched against a local CITES database that is retrieved from https://speciesplus.net. The final results are presented as a tab-separated values file (TSV) containing the BLAST hit metadata (i.e. bit-score, e-value, accession numbers etc.), the scientific name, synonym name, and in case a CITES-listed taxon was found, also the CITES appendix listing and taxonomic group (i.e. species, genus, family or order name) under which the taxon is listed by CITES.

The BLAST output was interpreted by following guidelines: first, to minimize the chance of erroneous species identifications, the same species should have at least three top hits, i.e. highest bit scores. Secondly, if multiple hits are obtained with identical quality results, but with different assigned species, or with less than three top hits with same species designation, the OTU fragment was considered to lack the discriminatory power to refer the hit to species level. In such cases, the OTU would then be downgraded to a genus-level identification. Thirdly, if multiple hits are obtained with identical quality results, but with different assigned genera, the OTU fragment lacks the discriminatory power to describe the hit to genus level. In such cases, the OTU would then be downgraded to a family-level identification. An online web-interface based application for the CITESspeciesDetect pipeline was developed which is available from http://decathlon-fp7.citespipe-wur.surf-hosted.nl:8080/. The web-interface facilitates intuitive BLAST identification of species listed by speciesplus.net by highlighting species on CITES appendix I in red. Species listed on CITES appendix II and II are highlighted in orange and yellow, respectively.

Pre-validation in-house of the CITESspeciesDetect pipeline

A parameter scan was performed in order to assess the effect of software settings on the ability to identify species. This evaluation allowed for identification of important parameters and their effects on the sensitivity, specificity and robustness of the procedure. This in turn resulted in specified, recommended (default) parameters values for analysing DNA metabarcoding datasets using the CITESspeciesDetect pipeline. The effects of the following parameters were assessed: base quality scores, error tolerance for primer selection, OTU radius, OTU abundance threshold, expect E-value and query coverage threshold, percentage identity threshold, minimum DNA barcode length and BLAST database. The parameters scan was performed on experimental mixture 11 of the pilot study (Table 2). This mixture was selected because of its (relatively) high sample complexity, making it the most challenging complex mixture to analyse. Furthermore, the parameter scan was limited to four barcode primer sets: full-length cytochrome-B (cyt *b*), COI mini barcode (mini-COI), *rbcL* mini barcode (mini-*rbcL)* and the full-length *rbcL* (*rbcL)* barcode.

Inter-laboratory validation trial: participants and method.

To assess the overall performance of the developed DNA metabarcoding approach, 16 laboratories from 11 countries participated in an international inter-laboratory validation. Only laboratories that regularly perform molecular analyses and have well-equipped laboratory facilities were selected to participate (Table 6). The majority are governmental or semi-official institutes and are considered highly authoritative within each respective country. Participants were requested to follow the SOP [21], and were asked to document any deviations that were made. The chemicals and reagents that were provided to the laboratories were: 10 samples (eight experimental mixtures and two TMs), *B. taurus* and *L. sativa* positive control DNA, CTAB extraction and precipitation buffer, 1.2 M NaCl solution, 12 universal plant and animal barcode and mini-barcode primer sets (Table 1), Qiagen HotStarTaq master mix, and Qiagen PCR purification kits. All reagents and samples were provided in quantities corresponding to 2.5× the amounts required for the planned experiments. After following the SOP from DNA isolation to purification of the amplified products, all the purified samples from all the laboratories (n=160) were collected and sequenced using Illumina MiSeq paired-end 300 technology (at BaseClear, Leiden, NL). The Index PCR and Illumina library preparation was performed according to the guideline and all 160 samples were sequenced on two Illumina flow cells. After Illumina MiSeq run, the raw NGS data was processed using the default settings of the CITESspeciesDetect pipeline. BLAST outputs for the

samples were distributed back to the participating laboratories for interpretation of results. The laboratories interpreted the BLAST output based on the guideline provided in the SOP.

Availability of supporting data

All the sequence data obtained from the pilot study and the international interlaboratory validation trial, the CITESspeciesDetect pipeline and access to web interface are freely available. The generated barcode sequence information for some animal and plant species were deposited in GenBank under the accession numbers LT009695 to LT009705, and LT718651 (Additional file 1; Table S1). The Illumina PE300 MiSeq data obtained from the pilot study and the international interlaboratory validation trial (n=177) were deposited to ENA with study ID PRJEB18620. The script for the CITESspeciesDetect pipeline is available at GitHub. The web interface for CITESspeciesDetect pipeline can be accessed via the following link: http://decathlon-fp7.citespipe-wur.surf-hosted.nl:8080/. The access to analysis via the web interface will be provided on request. SOP protocols are available from protocols.io [21] and snapshots of the code and example results are available from the *GigaScience* database[34].

Availability and requirements

Project name: CITESspeciesDetect

Project home page: https://github.com/RIKILT/CITESspeciesDetect

Operating system(s): Linux

Programming language: Python and Bash

Other requirements: none

License: BSD 3-Clause License

Any restrictions to use by non-academics: none

Additional files

Additional file 1: Table S1 Accession numbers of DNA barcode sequences of plant and animal species. **Table S2** PCR success rate for animal reference species. **Table S3** PCR success rate for plant reference species. **Table S4** Statistics of different quality filtering settings for four DNA barcodes. **Table S5** BLAST identification of species with different quality filtering settings for four DNA barcodes. **Table S6** Results of species-specific qPCR performed on the experimental mixtures prepared for the inter-laboratory validation trial. **Table S7** Interlaboratory trial study: average number of Illumina reads per sample, the average number of (pseudo)reads that passed quality control (QC) and the percentage of QC (pseudo)reads that were assigned to DNA barcodes and Operational Taxonomic Units (OTUs). **Table S8** qPCR primer and probe information. **Table S9** qPCR reagent composition. **Table S10** qPCR thermocycling program. (*.docx).

Additional file 2: Table S1 Pilot study: Composition of the experimental mixtures, and taxa identified using the default settings of the CITESspeciesDetect pipeline. **Table S2** Interlaboratory trial: *Beta vulgaris* observed in the sample S6 data sets generated by the 16 laboratories. **Table S3-S5** Interlaboratory trial: Assessment of the effect of different settings (OTU clusters size, OTU abundance threshold) of the CITESspeciesDetect pipeline on the identification of taxa using different data volume (low, medium and high) generated by three laboratory for S6. **Table S6** Interlaboratory trial: the taxonomic resolution provided by each DNA barcode marker for eight experimental mixtures (*.xlsx).

Additional file 3: Table S1 ENA accession numbers of all raw NGS datasets obtained in this study (*.xlsx).

Abbreviations

CITES: Convention on International trade in Endangered Species of Wild fauna and flora; TMs: Traditional Medicines; NGS: Next generation sequencing; CTAB: cetyltrimethylammonium bromide; COI: Cytochrome c oxidase subunit I; cyt *b*: Cytochrome *b* gene; 16S rDNA: 16S ribosomal DNA; *matK*: Maturase K gene; *rbcL*: ribulose-1,5-bisphosphate carboxylase large subunit gene; ITS2: Internal transcribed spacer region 2;; SOP: Standard operating procedure; OTU: Operational Taxonomic Unit; BLAST: Basic Local Alignment Search Tool.

Competing interests

The authors declare that they have no competing interest.

Funding

The DECATHLON project has been funded with support from the European Commission in the context of the Seventh Framework Programme (FP7). This publication and all its contents reflect the views only of the authors, and the Commission cannot be held responsible for any use, which may be made of the information contained therein.

Authors' Contributions

AJA and MS shared the first authorship. AJA, MS, MV, TP, AC, EK conceived and designed the experiments for the pilot study. AJA performed the experiments for the pilot study. MS, RH, AJA developed the CITESspeciesDetect pipeline. AJA, MS, RH analysed the NGS data obtained from the pilot study. AJA, MS, MV, TP, TWP, IS, EK, FG, MTBC, AHJ involved in establishing the Standard Operation Procedure for the validation trial. AJA, MS, MV, TP, EK conceived and designed the experiments for the validation trial. FG, MTBC, AHJ, AJA, MS involved in coordinating the trial. AJA, MV prepared the samples and materials for the validation trial and distributed to the participated laboratories. FR, MS, RH involved in developing the web-interface. MS, TP, DD, MBI, MBU, EH, RHO, AK, LL, CN, HN, EP, JPR, RS, TS, CVM took part in the validation trial. AJA, MS, RH, MV analysed the NGS data obtained from the validation trial. AJA, MS, RH, MV, SVR, EK contributed to the writing of the manuscript. All authors read and approved the final manuscript.

Acknowledgements

This work was supported by the DECATHLON project, which was funded by the European Commission under Seventh Framework Programme (FP7).

Reference:

1. Chang C-H, Jang-Liaw N-H, Lin Y-S, Fang Y-C, Shao K-T: **Authenticating the use of dried seahorses in the traditional Chinese medicine market in Taiwan using molecular forensics.** *Journal of Food and Drug Analysis* 2013, **21:**310-316.
2. Lee SY, Ng WL, Mahat MN, Nazre M, Mohamed R: **DNA Barcoding of the Endangered Aquilaria (Thymelaeaceae) and Its Application in Species Authentication of Agarwood Products Traded in the Market.** *PLOS One* 2016, **11:**e0154631.
3. Milner-Gulland E, Bukreeva O, Coulson T, Lushchekina A, Kholodova M, Bekenov A, Grachev IA: **Conservation: Reproductive collapse in saiga antelope harems.** *Nature* 2003, **422:**135-135.
4. Cheng X, Su X, Chen X, Zhao H, Bo C, Xu J, Bai H, Ning K: **Biological ingredient analysis of traditional Chinese medicine preparation based on high-throughput sequencing: the story for Liuwei Dihuang Wan.** *Scientific Reports* 2014, **4: 5147**.
5. Coghlan ML, Haile J, Houston J, Murray DC, White NE, Moolhuijzen P, Bellgard MI, Bunce M: **Deep sequencing of plant and animal DNA contained within traditional Chinese medicines reveals legality issues and health safety concerns.** *PLOS Genetics* 2012, **8:**e1002657.

6. Coghlan ML, Maker G, Crighton E, Haile J, Murray DC, White NE, Byard RW, Bellgard MI, Mullaney I, Trengove R: **Combined DNA, toxicological and heavy metal analyses provides an auditing toolkit to improve pharmacovigilance of traditional Chinese medicine (TCM).** *Scientific Reports* 2015, **5**.
7. Ivanova NV, Kuzmina ML, Braukmann TWA, Borisenko AV, Zakharov EV: **Authentication of Herbal Supplements Using Next-Generation Sequencing.** *PLOS One* 2016, **11:**e0156426.
8. Taberlet P, Coissac E, Pompanon F, Brochmann C, Willerslev E: **Towards next-generation biodiversity assessment using DNA metabarcoding.** *Molecular Ecology* 2012, **21:**2045-2050.
9. Staats M, Arulandhu AJ, Gravendeel B, Holst-Jensen A, Scholtens I, Peelen T, Prins TW, Kok E: **Advances in DNA metabarcoding for food and wildlife forensic species identification.** *Analytical and Bioanalytical Chemistry* 2016**:**1-16.
10. Fahner NA, Shokralla S, Baird DJ, Hajibabaei M: **Large-scale monitoring of plants through environmental DNA metabarcoding of soil: recovery, resolution, and annotation of four DNA markers.** *PLOS One* 2016, **11:**e0157505.
11. Arulandhu AJ, Staats M, Peelen T, Kok E: **DNA metabarcoding of endangered plant and animal species in seized forensic samples.** In *Genome*. 2015: 188-189.
12. Taylor H, Harris W: **An emergent science on the brink of irrelevance: a review of the past 8 years of DNA barcoding.** *Molecular Ecology Resources* 2012, **12:**377-388.
13. Little DP: **A DNA mini-barcode for land plants.** *Molecular Ecology Resources* 2014, **14:**437-446.
14. Parveen I, Gafner S, Techen N, Murch SJ, Khan IA: **DNA Barcoding for the Identification of Botanicals in Herbal Medicine and Dietary Supplements: Strengths and Limitations.** *Planta Medica* 2016, **82:**1225-1235.
15. Chen R, Dong J, Cui X, Wang W, Yasmeen A, Deng Y, Zeng X, Tang Z: **DNA based identification of medicinal materials in Chinese patent medicines.** *Scientific Reports* 2012, **2:**958.
16. Scholtens I, Laurensse E, Molenaar B, Zaaijer S, Gaballo H, Boleij P, Bak A, Kok E: **Practical experiences with an extended screening strategy for genetically modified organisms (GMOs) in real-life samples.** *Journal of agricultural and food chemistry* 2013, **61:**9097-9109.
17. Murray M, Thompson WF: **Rapid isolation of high molecular weight plant DNA.** *Nucleic Acids Research* 1980, **8:**4321-4326.
18. Ivanova NV, Zemlak TS, Hanner RH, Hebert PDN: **Universal primer cocktails for fish DNA barcoding.** *Molecular Ecology Notes* 2007, **7:**544-548.
19. Edgar RC: **Search and clustering orders of magnitude faster than BLAST.** *Bioinformatics* 2010, **26:**2460-2461.
20. Altschul SF, Madden TL, Schäffer AA, Zhang J, Zhang Z, Miller W, Lipman DJ: **Gapped BLAST and PSI-BLAST: a new generation of protein database search programs.** *Nucleic Acids Research* 1997, **25:**3389-3402.
21. Alfred J. Arulandhu, Martijn Staats, Rico Hagelaar, Marleen M. Voorhuijzen, Theo W. Prins, Ingrid Scholtens, Tamara Peelen and Esther Kok (2017): Development and validation of a multi-locus DNA metabarcoding method to identify endangered species in complex samples SOP. protocols.io. http://dx.doi.org/10.17504/protocols.io.ixbcfin
22. Hebert PD, Cywinska A, Ball SL, deWaard JR: **Biological identifications through DNA barcodes.** *Proceedings of the Royal Society of London B: Biological Sciences* 2003, **270:**313-321.
23. Group CPW, Hollingsworth PM, Forrest LL, Spouge JL, Hajibabaei M, Ratnasingham S, van der Bank M, Chase MW, Cowan RS, Erickson DL: **A DNA barcode for land plants.** *Proceedings of the National Academy of Sciences* 2009, **106:**12794-12797.
24. Liu S, Li Y, Lu J, Su X, Tang M, Zhang R, Zhou L, Zhou C, Yang Q, Ji Y: **SOAPBarcode: revealing arthropod biodiversity through assembly of Illumina shotgun sequences of PCR amplicons.** *Methods in Ecology and Evolution* 2013, **4:**1142-1150.
25. Gryson N: **Effect of food processing on plant DNA degradation and PCR-based GMO analysis: a review.** *Analytical and Bioanalytical Chemistry* 2010, **396:**2003-2022.
26. Tang M, Hardman CJ, Ji Y, Meng G, Liu S, Tan M, Yang S, Moss ED, Wang J, Yang C: **High‐throughput monitoring of wild bee diversity and abundance via mitogenomics.** *Methods in Ecology and Evolution* 2015, **6:**1034-1043.
27. Galimberti A, De Mattia F, Losa A, Bruni I, Federici S, Casiraghi M, Martellos S, Labra M: **DNA barcoding as a new tool for food traceability.** *Food Research International* 2013, **50:**55-63.
28. Taberlet P, Coissac E, Hajibabaei M, Rieseberg LH: **Environmental DNA.** *Molecular Ecology* 2012, **21:**1789-1793.
29. Iyengar A: **Forensic DNA analysis for animal protection and biodiversity conservation: a review.** *Journal for Nature Conservation* 2014, **22:**195-205.

30. **16s Metagenomic Sequencing Library Preparation. Illumina document 15044223.** https://support.illumina.com/content/dam/illumina-support/documents/documentation/chemistry_documentation/16s/16s-metagenomic-library-prep-guide-15044223-b.pdf
31. Martin M: **Cutadapt removes adapter sequences from high-throughput sequencing reads.** *EMBnet journal* 2011, **17:**10-12.
32. Schmieder R, Edwards R: **Quality control and preprocessing of metagenomic datasets.** *Bioinformatics* 2011, **27:**863-864.
33. **FASTX_Toolkit v0.0.14** http://hannonlab.cshl.edu/fastx_toolkit/
34. Arulandhu, A, J; Hagelaar, R; Staats, M; Voorhuijzen, M, M; Prins, T, W; Scholtens, I, M; Costessi, A; Duijsings, D; Rechenmann, F; Gaspar, F, B; Barreto Crespo, M, T; Holst-Jensen, A; Birck, M; Burns, M; Haynes, E; Hochegger, R; Klingl, A; Lundberg, L; Natale, C; Niekamp, H; Perri, E; Barbante, A; Rosec, J; Seyfarth, R; Sovova, T; Moorleghem, C, V; Ruth, S, V; Peelen, T; Kok, E (2017): Supporting data for "Development and validation of a multi-locus DNA metabarcoding method to identify endangered species in complex samples" GigaScience Database. http://dx.doi.org/10.5524/100330
35. Palumbi S, Martin A, Romano S, McMillan W, Stice L, Grabowski G: **The Simple Fool's Guide to PCR, Version 2.0, privately published document compiled by S. Palumbi. Dept.** *Zoology, Univ Hawaii, Honolulu, HI* 1991, **96822**.
36. Sarri C, Stamatis C, Sarafidou T, Galara I, Godosopoulos V, Kolovos M, Liakou C, Tastsoglou S, Mamuris Z: **A new set of 16S rRNA universal primers for identification of animal species.** *Food Control* 2014, **43:**35-41.
37. Leray M, Yang JY, Meyer CP, Mills SC, Agudelo N, Ranwez V, Boehm JT, Machida RJ: **A new versatile primer set targeting a short fragment of the mitochondrial COI region for metabarcoding metazoan diversity: application for characterizing coral reef fish gut contents.** *Front Zool* 2013, **10:**34.
38. Geller J, Meyer C, Parker M, Hawk H: **Redesign of PCR primers for mitochondrial cytochrome *c* oxidase subunit I for marine invertebrates and application in all-taxa biotic surveys.** *Molecular Ecology Resources* 2013, **13:**851-861.
39. Parson W, Pegoraro K, Niederstätter H, Föger M, Steinlechner M: **Species identification by means of the cytochrome *b* gene.** *International Journal of Legal Medicine* 2000, **114:**23-28.
40. Fazekas AJ, Kuzmina ML, Newmaster SG, Hollingsworth PM: **DNA barcoding methods for land 39.** *Methods in Molecular Biology* 2012, **858:**223-252.
41. Cuénoud P, Savolainen V, Chatrou LW, Powell M, Grayer RJ, Chase MW: **Molecular phylogenetics of Caryophyllales based on nuclear 18S rDNA and plastid *rbcL*, *atpB*, and *matK* DNA sequences.** *American Journal of Botany* 2002, **89:**132-144.
42. Levin RA, Wagner WL, Hoch PC, Nepokroeff M, Pires JC, Zimmer EA, Sytsma KJ: **Family-level relationships of Onagraceae based on chloroplast *rbcL* and *ndhF* data.** *American Journal of Botany* 2003, **90:**107-115.
43. Kress WJ, Erickson DL: **A two-locus global DNA barcode for land plants: the coding *rbcL* gene complements the non-coding *trnH-psbA* spacer region.** *PLOS One* 2007, **2:**e508.
44. Fazekas AJ, Burgess KS, Kesanakurti PR, Graham SW, Newmaster SG, Husband BC, Percy DM, Hajibabaei M, Barrett SC: **Multiple multilocus DNA barcodes from the plastid genome discriminate plant species equally well.** *PLOS One* 2008, **3:**e2802.
45. Taberlet P, Coissac E, Pompanon F, Gielly L, Miquel C, Valentini A, Vermat T, Corthier G, Brochmann C, Willerslev E: **Power and limitations of the chloroplast trnL (UAA) intron for plant DNA barcoding.** *Nucleic Acids Research* 2007, **35:**e14.
46. Chen S, Yao H, Han J, Liu C, Song J, Shi L, Zhu Y, Ma X, Gao T, Pang X: **Validation of the ITS2 region as a novel DNA barcode for identifying medicinal plant species.** *PLOS One* 2010, **5:**e8613.
47. Sang T, Crawford D, Stuessy T: **Chloroplast DNA phylogeny, reticulate evolution, and biogeography of *Paeonia* (Paeoniaceae).** *American Journal of Botany* 1997, **84:**1120-1136.
48. Tate JA, Simpson BB: **Paraphyly of *Tarasa* (Malvaceae) and diverse origins of the polyploid species.** *Systematic Botany* 2003, **28:**723-737.
49. Manning J, Boatwright JS, Daru BH, Maurin O, Bank Mvd: **A molecular phylogeny and generic classification of Asphodelaceae subfamily Alooideae: a final resolution of the prickly issue of polyphyly in the alooids?** *Systematic Botany* 2014, **39:**55-74.

Figure Click here to download Figure A multi-locus DNA metabarcoding method_Fig_AJ_resubmission.docx

Figure file:

Development and validation trial of a multi-locus DNA metabarcoding method to identify endangered species in complex samples.

Alfred J. Arulandhu, Martijn Staats, Rico Hagelaar, Marleen M. Voorhuijzen, Theo W. Prins, Ingrid Scholtens, Adalberto Costessi, Danny Duijsings, François Rechenmann, Frédéric B. Gaspar, Maria Teresa Barreto Crespo, Arne Holst-Jensen, Matthew Birck, Malcolm Burns, Edward Haynes , Rupert Hochegger, Alexander Klingl, Lisa Lundberg, Chiara Natale , Hauke Niekamp, Elena Perri, Alessandra Barbante , Jean-Philippe Rosec, Ralf Seyfarth, Tereza Sovová, Christoff Van Moorleghem, Saskia van Ruth, Tamara Peelen and Esther Kok

Figure 1: Schematic representation of the CITESspeciesDetect pipeline.

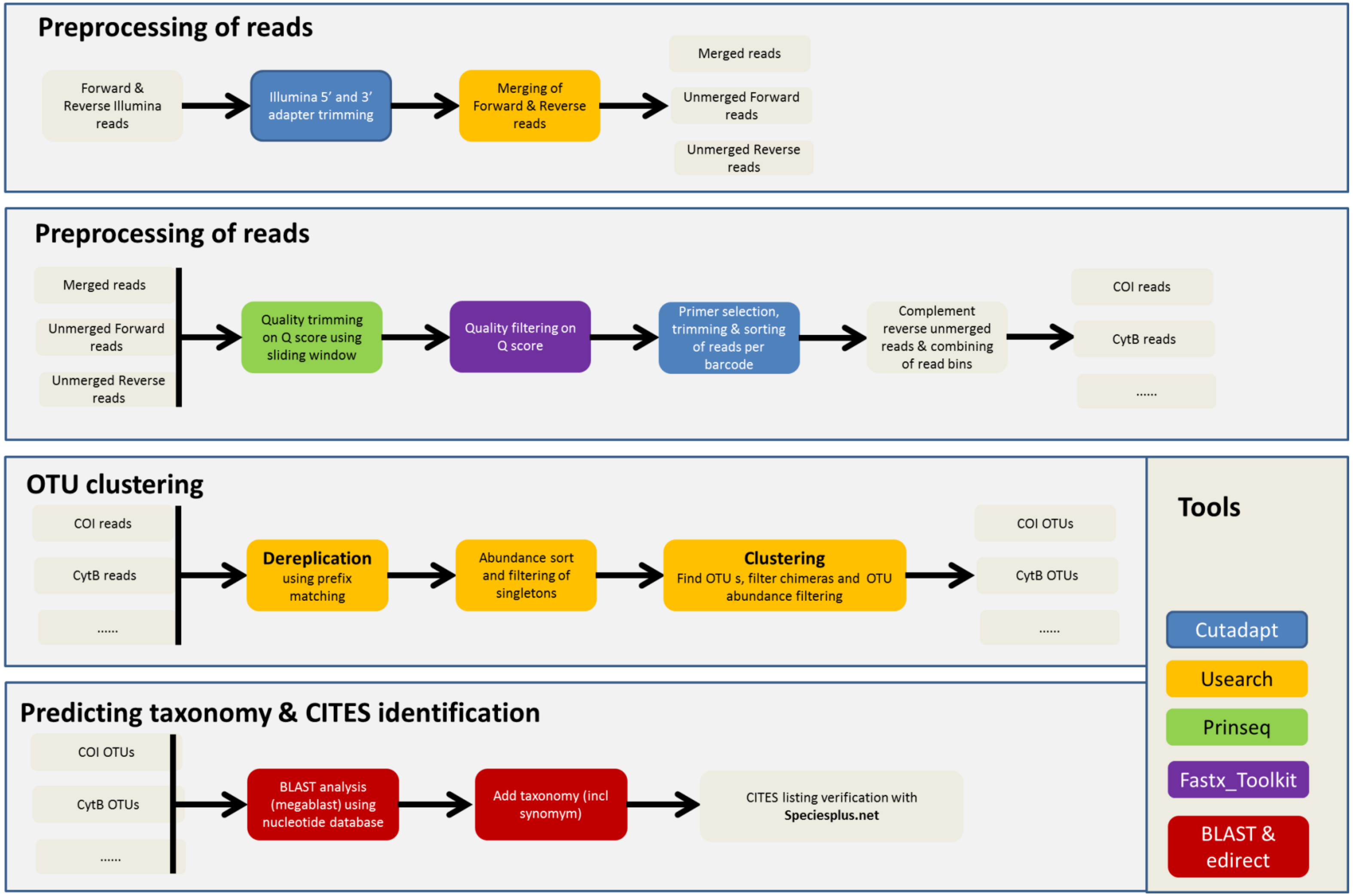

Figure 2: The percentage of QC reads assigned to DNA barcodes for samples EM10, EM10R, EM11 and EM11R of the pilot study.

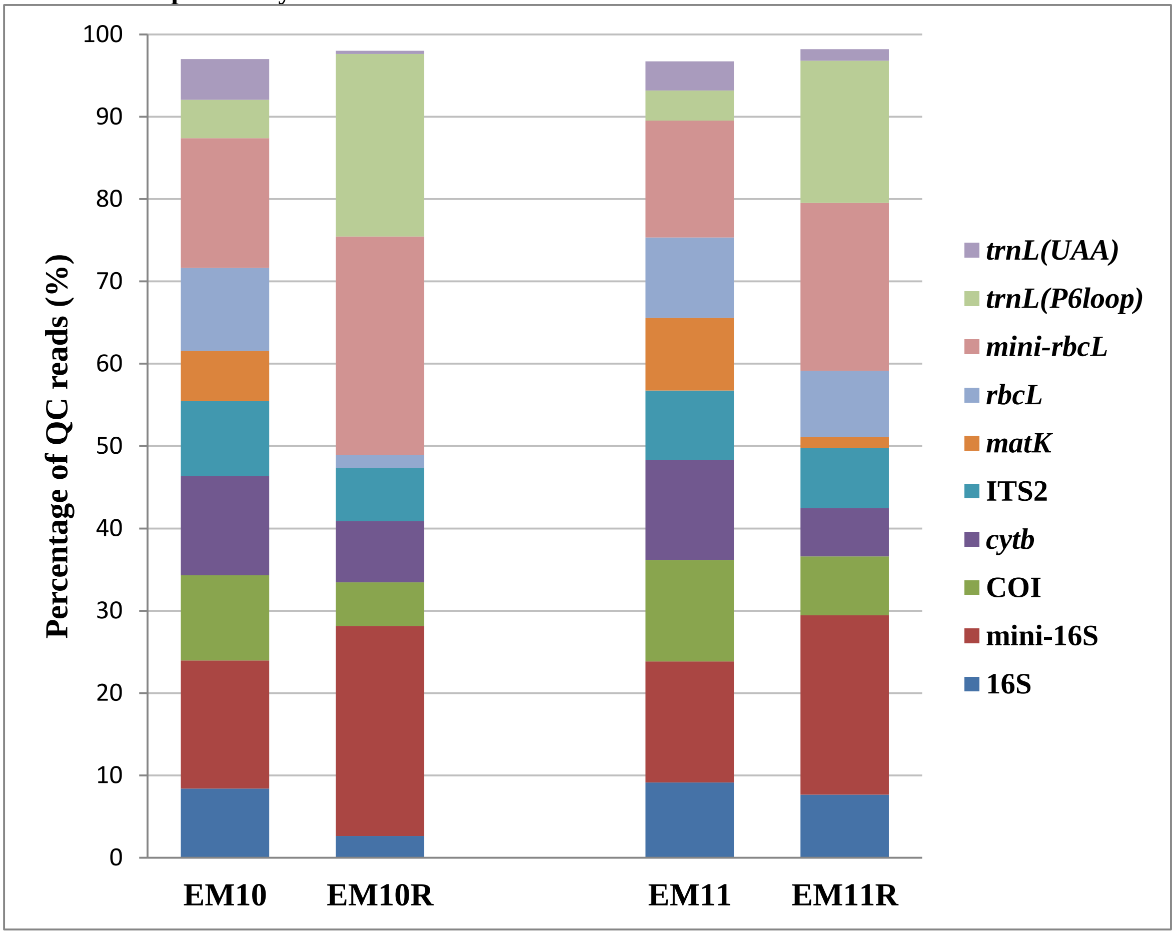

Click here to access/download
Supplementary Material
A multi-locus DNA metabarcoding method_Tab_AJ_resubmission.docx

Click here to access/download
Supplementary Material
Additional file 1_Table S1-S10_Gigascience_AJ_resubmission.docx

Click here to access/download
Supplementary Material
Additional files 2_ Table S1-S2_Gigascience_AJ_resubmission.xlsx

Click here to access/download
Supplementary Material
Additional file 3_Gigascience_AJ_resubmission.xlsx
