# Supplementary material for: Development and validation of a multi-locus DNA metabarcoding method to identify endangered species in complex samples
Source: Gigascience. 2017 Aug 19;6(10):1–18. doi: 10.1093/gigascience/gix080 (PMC5632295; doi:10.1093/gigascience/gix080)
Supplement: Additional Files [file gix080_Supp.zip › Additional file 1_Table S1-S10_Gigascience_AJ_resubmission.docx]

**Additional file 1 of:**

**Development and validation trial of a multi-locus DNA metabarcoding method to identify endangered species in complex samples.**

Alfred J. Arulandhu, Martijn Staats, Rico Hagelaar, Marleen M. Voorhuijzen, Theo W. Prins, Ingrid Scholtens, Adalberto Costessi, Danny Duijsings, François Rechenmann, Frédéric B. Gaspar, Maria Teresa Barreto Crespo, Arne Holst-Jensen, Matthew Birck, Malcolm Burns, Edward Haynes , Rupert Hochegger, Alexander Klingl, Lisa Lundberg, Chiara Natale , Hauke Niekamp, Elena Perri, Alessandra Barbante , Jean-Philippe Rosec, Ralf Seyfarth, Tereza Sovová, Christoff Van Moorleghem, Saskia van Ruth, Tamara Peelen and Esther Kok

**Table S1: Accession numbers of DNA barcode sequences of plants and animal species.**

| **Accession No.** | **Description** |
| --- | --- |
| LT009695 | *Parapenaeopsis* sp. MS-2016 partial 16S rRNA gene |
| LT009696 | *Parapenaeopsis* sp. MS-2016 mitochondrial partial COI gene for cytochrome oxidase subunit 1 |
| LT009697 | *Gonialoe variegata* genomic DNA sequence contains 5.8S rRNA gene, ITS2, 28S rRNA gene |
| LT009698 | *Echinocactus* sp. MS-2016 genomic DNA sequence contains 5.8S rRNA gene, ITS2, 28S rRNA gene |
| LT009699 | *Cycas revoluta* genomic DNA sequence contains 5.8S rRNA gene, ITS2, 28S rRNA gene |
| LT009700 | *Cycas revoluta* genomic DNA sequence contains 5.8S rRNA gene, ITS2, 28S rRNA gene |
| LT009701 | *Euphorbia* sp. MS-2016 genomic DNA sequence contains 5.8S rRNA gene, ITS2, 28S rRNA gene |
| LT009702 | *Lactuca sativa* genomic DNA sequence contains 5.8S rRNA gene, ITS2, 28S rRNA gene |
| LT009703 | *Echinocactus* sp. MS-2016 chloroplast partial *rbcL* gene for ribulose 1,5-bisphosphate carboxylase large subunit |
| LT009704 | *Dendrobium* sp. MS-2016 chloroplast partial *rbcL* gene for ribulose 1,5-bisphosphate carboxylase large subunit |
| LT009705 | *Euphorbia* sp. MS-2016 chloroplast partial *rbcL* gene for ribulose 1,5-biphosphate carboxylase large subunit |
| LT718651 | *Brassica napus* genomic DNA sequence contains 5.8S rRNA gene, ITS2, and 28S rRNA gene, strain MS-2016 |

**Table S2: PCR success rate for animal reference species.**

| **Scientific name** | **Common name** | **Barcode markers** | | | **Mini-barcode markers** | | |
| --- | --- | --- | --- | --- | --- | --- | --- |
|  |  | ***16S*** | ***COI*** | **cyt *b*** | ***16S*** | ***COI*** | **cyt *b*** |
| **Mammals (n=9)** |  |  |  |  |  |  |  |
| *Bos taurus* | Cattle | ✓✓ | ✓✓ | ✓✓ | ✓✓ | ✓✓ | ✓✓ |
| *Equus ferus* | Horse | ✓✓ | ✓✓ | ✓✓ | ✓✓ | ✓✓ | ✓✓ |
| *Macropus* sp*.* | kangaroo | ✓✓ | ✓✓ | ✓✓ | ✓✓ | ✓✓ | ✓✓ |
| *Leporidae* sp*.* | Rabbit | ✓✓ | ✓✓ | ✓✓ | ✓✓ | ✓✓ | ✓✓ |
| *Homo sapiens* | Human | ✓✓ | 🗶 | ✓ | ✓✓ | ✓✓ | ✓ |
| *Canis lupus* | Dog | ✓✓ | 🗶 | 🗶 | ✓✓ | ✓✓ | ✓ |
| *Sus scrofa* | Pig | ✓✓ | ✓✓ | ✓✓ | ✓✓ | ✓✓ | ✓✓ |
| *Ursus arctos** | Brown bear | ✓✓ | ✓✓ | ✓✓ | ✓✓ | ✓✓ | ✓✓ |
| *Balaenoptera physalus** | Fine whale | ✓✓ | ✓✓ | 🗶 | ✓✓ | ✓✓ | ✓ |
| **Fishes (n=9)** |  |  |  |  |  |  |  |
| *Rutilus rutilus* | Roach | ✓✓ | ✓ | ✓✓ | ✓✓ | ✓✓ | ✓✓ |
| *Thunnus albacares* | Yellowfin tuna | ✓✓ | ✓✓ | ✓✓ | ✓✓ | ✓✓ | ✓✓ |
| *Pangasius hypophthalmus* | Iridescent shark | ✓✓ | 🗶 | ✓✓ | ✓✓ | ✓✓ | ✓✓ |
| *Huso dauricus** | Kaluga | ✓✓ | ✓✓ | ✓✓ | ✓✓ | ✓✓ | ✓✓ |
| *Anguilla anguilla** | European eel | ✓✓ | ✓ | ✓ | ✓✓ | ✓✓ | ✓✓ |
| *Salmo salar* | Atlantic salmon | ✓✓ | ✓✓ | ✓✓ | ✓✓ | ✓✓ | ✓✓ |
| *Pleuronectes platessa* | European plaice | ✓✓ | 🗶 | 🗶 | ✓✓ | ✓✓ | ✓✓ |
| *Parapenaeopsis* sp*.* | Shrimp | ✓✓ | 🗶 | 🗶 | ✓✓ | ✓✓ | ✓ |
| *Mytilus trossulus* | Bay mussel | 🗶 | 🗶 | 🗶 | 🗶 | ✓✓ | 🗶 |
| **Aves (n=6)** |  |  |  |  |  |  |  |
| *Gallus gallus* | Chicken | ✓✓ | ✓✓ | ✓✓ | ✓✓ | ✓✓ | ✓✓ |
| *Cairina moschata* | Muscovy duck | ✓✓ | ✓✓ | ✓✓ | ✓✓ | ✓✓ | ✓✓ |
| *Columba palumbus* | Wood pigeon | ✓✓ | ✓✓ | ✓ | ✓✓ | ✓✓ | ✓✓ |
| *Larus argentatus* | European herring gull | ✓✓ | ✓✓ | ✓✓ | ✓✓ | ✓✓ | ✓✓ |
| *Meleagris gallopavo* | Turkey | ✓✓ | ✓✓ | ✓✓ | ✓✓ | ✓✓ | ✓✓ |
| *Sturnus vulgaris* | Starling | ✓✓ | ✓✓ | ✓✓ | ✓✓ | ✓✓ | ✓✓ |
| **Reptiles (n=2)** |  |  |  |  |  |  |  |
| *Crocodylus porosus** | Saltwater crocodile | ✓✓ | 🗶 | ✓✓ | ✓✓ | ✓✓ | ✓✓ |
| *Crocodylus Niloticus** | Nile crocodile | ✓✓ | ✓ | ✓✓ | ✓✓ | ✓✓ | ✓✓ |
| **Amphibians (n=2)** |  |  |  |  |  |  |  |
| *Fejervarya cancrivora* | Crab-eating frog | ✓✓ | 🗶 | 🗶 | ✓✓ | ✓✓ | ✓ |
| *Hoplobatrachus rugulosus* | Chinese edible frog | ✓✓ | 🗶 | 🗶 | ✓✓ | ✓✓ | ✓ |
| **Insect (n=1)** |  |  |  |  |  |  |  |
| *Pieris brassicae* | Large white | ✓ | ✓✓ | 🗶 | ✓✓ | ✓✓ | ✓ |

An asterisk (*) indicates a species listed by CITES. The Symbols (✓✓) and (✓) indicate the intensity of the specific amplification for the target species. The symbol (🗶) indicates no PCR amplification.

**Table S3: PCR success rate for plant reference species.**

| **Scientific name** | | **Common name** | **Barcode markers** | | | **Mini-barcode markers** | | |
| --- | --- | --- | --- | --- | --- | --- | --- | --- |
|  |  |  | ***matK*** | ***rbcL*** | ***trnL*** | **ITS2** | ***rbcL*** | ***trnL*** |
| *Echinocactus* sp*.** | | Barrel cactus | ✓✓ | ✓✓ | ✓ | ✓✓ | ✓✓ | ✓✓ |
| *Euphorbia* sp*.** | | Spurge | ✓✓ | ✓✓ | ✓ | ✓✓ | ✓✓ | ✓✓ |
| *Phaseolus vulgaris* | | Bean | ✓✓ | ✓✓ | ✓ | ✓✓ | ✓ ✓ | ✓✓ |
| *Aloe variegata** | | Tiger aloe | ✓✓ | ✓✓ | ✓ | ✓✓ | ✓✓ | ✓✓ |
| *Dendrobium* sp*.** | | Dendrobium orchid | ✓✓ | ✓✓ | ✓ | ✓✓ | ✓✓ | ✓✓ |
| *Dypsis lutescens ** | | Areca palm | ✓✓ | ✓✓ | ✓ | ✓✓ | ✓✓ | ✓✓ |
| *Cycas revoluta** | | Sago palm | ✓✓ | ✓✓ | ✓ | ✓✓ | ✓✓ | ✓✓ |
| *Lactuca sativa* | | Lettuce | ✓✓ | ✓✓ | ✓ | ✓✓ | ✓✓ | ✓✓ |
| *Glycine max* | | Soy bean | ✓✓ | ✓✓ | ✓ | ✓✓ | ✓✓ | ✓✓ |
| *Gossypium hirsutum* | | Cotton | ✓✓ | ✓✓ | ✓ | ✓✓ | ✓✓ | ✓✓ |
| *Brassica napus* | | Canola | ✓✓ | ✓✓ | ✓ | ✓✓ | ✓✓ | ✓✓ |
| *Triticum aestivum* | | Wheat | ✓✓ | ✓✓ | ✓ | ✓✓ | ✓✓ | ✓✓ |
| *Beta vulgaris* | | Sugar beet | ✓✓ | ✓✓ | ✓ | ✓✓ | ✓✓ | ✓✓ |
| *Brassica oleracea* | | White cabbage | ✓✓ | ✓✓ | ✓ | ✓✓ | ✓✓ | ✓✓ |
| *Carica papaya* | | Papaya | ✓✓ | ✓✓ | ✓ | ✓✓ | ✓✓ | ✓✓ |
| *Solanum lycopersicum* | | Tomato | ✓✓ | ✓✓ | ✓ | ✓✓ | ✓✓ | ✓✓ |
| *Zea mays* | | Maize | ✓✓ | ✓✓ | ✓ | ✓✓ | ✓✓ | ✓✓ |
|  |  | | | | | | | |

An asterisk (*) indicates a species listed by CITES. The Symbols (✓✓) and (✓) indicate the intensity of the specific amplification for the target species. The symbol (🗶) indicates no PCR amplification.

**Table S4: Statistics of different quality filtering settings for four DNA barcodes. Default setting is marked grey.**

|  |  | **Q10_95%** | **Q10_99%** | **Q20_95%** | **Q20_99%** | **Q20_100%** | **Q30_95%** | **Q30_99%** |
| --- | --- | --- | --- | --- | --- | --- | --- | --- |
| ***cyt*B** | # PS merged: | 16881 | 16124 | 16472 | 11471 | 2427 | 8240 | 67 |
|  | # PS FW: | 6650 | 6533 | 6258 | 2741 | 1004 | 2285 | 132 |
|  | # PS RV: | 4943 | 4592 | 3128 | 1113 | 671 | 702 | 89 |
|  | # combined file: | 28474 | 27249 | 25858 | 15325 | 4102 | 11227 | 288 |
|  | # Sorted by length: | 28474 | 27249 | 25858 | 15325 | 4102 | 11227 | 288 |
|  | # Dereplication: | 8597 | 7649 | 6741 | 3189 | 1157 | 2474 | 123 |
|  | # Derep clusters: | 707 | 694 | 667 | 405 | 95 | 319 | 11 |
|  | # After clustering: | 28 | 29 | 20 | 15 | 11 | 8 | 4 |
|  | # Trimmed clusters: | 28 | 29 | 20 | 15 | 11 | 8 | 4 |
| **mini-COI*** | # PS merged: | 16715 | 15957 | 16253 | 11960 | 3435 | 10216 | 304 |
|  | # PS FW: | 1612 | 1579 | 1552 | 957 | 410 | 791 | 110 |
|  | # PS RV: | 0 | 0 | 0 | 0 | 0 | 0 | 0 |
|  | # combined file: | 18327 | 17536 | 17805 | 12917 | 3845 | 11007 | 414 |
|  | # Sorted by length: | 18327 | 17536 | 17805 | 12917 | 3845 | 11007 | 414 |
|  | # Dereplication: | 8768 | 8087 | 8297 | 5678 | 1711 | 4884 | 213 |
|  | # Derep clusters: | 274 | 274 | 275 | 197 | 74 | 160 | 16 |
|  | # After clustering: | 4 | 7 | 5 | 3 | 2 | 4 | 2 |
|  | # Trimmed clusters: | 4 | 7 | 5 | 3 | 2 | 4 | 2 |
| **mini-*rbcL*** | # PS merged: | 24358 | 24298 | 24346 | 24126 | 22982 | 24272 | 22705 |
|  | # PS FW: | 4626 | 4611 | 4442 | 3804 | 3191 | 3747 | 2341 |
|  | # PS RV: | 3591 | 3588 | 3186 | 2111 | 1511 | 1881 | 345 |
|  | # combined file: | 32575 | 32497 | 31974 | 30041 | 27684 | 29900 | 25391 |
|  | # Sorted by length: | 32575 | 32497 | 31974 | 30041 | 27684 | 29900 | 25391 |
|  | # Dereplication: | 2304 | 2270 | 1944 | 1463 | 1224 | 1458 | 1002 |
|  | # Derep clusters: | 309 | 311 | 305 | 286 | 263 | 277 | 244 |
|  | # After clustering: | 18 | 18 | 19 | 17 | 16 | 18 | 15 |
|  | # Trimmed clusters: | 18 | 18 | 19 | 17 | 16 | 18 | 15 |
| ***rbcL*** | # PS merged: | 1484 | 1482 | 1483 | 1464 | 1218 | 1468 | 1230 |
|  | # PS FW: | 11098 | 10991 | 10718 | 8259 | 4465 | 7448 | 1378 |
|  | # PS RV: | 9866 | 9853 | 8622 | 4396 | 2478 | 3021 | 180 |
|  | # combined file: | 22448 | 22326 | 20823 | 14119 | 8161 | 11937 | 2788 |
|  | # Sorted by length: | 22448 | 22326 | 20823 | 14119 | 8161 | 11937 | 2788 |
|  | # Dereplication: | 5388 | 5336 | 4382 | 2539 | 1567 | 2187 | 737 |
|  | # Derep clusters: | 456 | 449 | 436 | 304 | 182 | 261 | 57 |
|  | # After clustering: | 22 | 21 | 22 | 21 | 16 | 15 | 8 |
|  | # Trimmed clusters: | 22 | 21 | 22 | 21 | 16 | 15 | 8 |

An asterisk (*) indicates the combined reads from full-length COI and mini-COI.

**Table S5. BLAST identification of species with different quality filtering settings for four DNA barcodes. Taxa were identified at the species, genus, family or order level.**

|  | **Q10_95%** | **Q10_99%** | **Q20_95%** | **Q20_99%** | **Q20_100%** | **Q30_95%** | **Q30_99%** |
| --- | --- | --- | --- | --- | --- | --- | --- |
| **cyt *b*** | *Bos taurus* (2x) | *Bos taurus* (2x) | *Bos taurus* (3x) | *Bos taurus* (3x) | *Bos taurus* | *Bos taurus* (2x) | *Bos taurus* |
|  | *Gallus gallus* | *Gallus gallus* | *Gallus gallus* | *Gallus gallus* | *Gallus gallus* | *Gallus gallus* | *Gallus gallus* |
|  | *Bos* | *Bos* | *Bos* | *Bos* | *Bos* | *Bos* |  |
|  | *Anguilla anguilla* | *Anguilla anguilla* | *Anguilla anguilla* | *Anguilla anguilla* | *Anguilla anguilla* | *Anguilla anguilla* |  |
| **mini-COI*** | *Bos taurus* | *Bos taurus* (4x) | *Bos taurus* (4x) | *Bos taurus* (2x) | *Bos taurus* (2x) | *Bos taurus* (2x) | *Bos taurus* (2x) |
|  | *Pieris brassicae* | *Pieris brassicae* | *Pieris brassicae* | *Pieris brassicae* |  | *Pieris brassicae* |  |
| **mini-*rbcL*** | Asteraceae (2x) | Asteraceae (3x) | Asteraceae (3x) | Asteraceae (3x) | Asteraceae (2x) | Asteraceae (3x) | Asteraceae (2x) |
|  | Xanthorrhoeaceae | Xanthorrhoeaceae | Xanthorrhoeaceae | Xanthorrhoeaceae | Xanthorrhoeaceae | Xanthorrhoeaceae | Xanthorrhoeaceae |
| ***rbcL*** | Asteraceae (3x) | Asteraceae (3x) | Asteraceae (3x) | Asteraceae (3x) | Asteraceae (3x) | Asteraceae (3x) | Asteraceae (3x) |
|  | Xanthorrhoeaceae | Xanthorrhoeaceae | Xanthorrhoeaceae | Xanthorrhoeaceae | Xanthorrhoeaceae | Xanthorrhoeaceae | Xanthorrhoeaceae |
|  | Cactaceae | Cactaceae | Cactaceae | Cactaceae | Cactaceae | Cactaceae | Cactaceae |
|  | Euphorbia | Euphorbia | Euphorbia | Euphorbia | Euphorbia | Euphorbia | Euphorbia |

The number of OTUs with which each taxon is identified is provided in brackets. Default setting is marked grey. An asterisk (*) indicates combined BLAST output from full-length COI and mini-COI.

**Table S6: Verifying the homogeneity and sample cross-contamination in the samples prepared for inter-laboratory study.**

|  |  | **Homogenized mixtures** | | | | | | | |
| --- | --- | --- | --- | --- | --- | --- | --- | --- | --- |
| **Species name** | **qPCR target** | **S1** | **S2** | **S4** | **S5** | **S6** | **S7** | **S9** | **S10** |
| *Zea mays §* | *HMG* | ✓ | ✓ | ✓ | ✓ | ✓ | ✓ | ✓ | ✓ |
| *Glycine max ǂ* | *Le1* | ✓ |  |  |  |  |  |  |  |
| *Gossypium* sp*. ǂ* | *acp1* |  | ✓ |  |  |  |  |  |  |
| *Brassica napus ǂ* | *FatA* | ✓ | ✓ | ✓ | ✓ | ✓ | ✓ | ✓ | ✓ |
| *Triticum aestivum ǂ* | *Wx-1* |  |  |  | ✓ |  |  |  |  |
| *Beta vulgaris ǂ* | *GS* |  |  |  |  | ✓ |  |  |  |
| *Meleagris gallopavo ǂ* | *melIL* |  |  |  |  |  | ✓ |  |  |
| *Carica papaya ǂ* | CHY |  |  |  |  |  |  | ✓ |  |
| *Solanum lycopersicum ǂ* | LAT52 |  |  |  |  |  |  |  | ✓ |

The symbol (§) indicates the species used to check the homogeneity of the sample and the symbol (ǂ) indicates the species used to identify the sample cross-contamination with in the samples. The symbol (✓) indicates the detection of the species in the mixture using species specific qPCR assay.

**Table S7: Average number of Illumina MiSeq reads per sample generated by BaseClear, the average number of (pseudo)reads that passed quality control (QC) and the percentage of QC (pseudo)reads that were assigned to DNA barcodes and Operational Taxonomic Units (OTUs).**

| Sample | Average number of raw reads  [min -max] | Average number of QC (pseudo)reads^*^  [min – max] | Percentage DNA barcode assigned (pseudo)reads^*^  [min – max] | Percentage OTU assigned (pseudo)reads^*^  [min – max] |
| --- | --- | --- | --- | --- |
| Sample_1 (S1) | 212,904  [164,250 – 338,374] | 114,772  [89,956 – 175,858] | 97.06  [96.46 - 97.97] | 80.05  [74.93 – 88.84] |
| Sample_2 (S2) | 197,855  [102,476 – 361,246] | 106,275  [52,553 – 189,351] | 96.83  [93.30 - 97.79] | 78.90  [70.49 – 88.55] |
| Sample_3 (S3)^#^ | 277,955  [201,762 – 463,802] | 143,395  [105,421 – 231,497] | 94.38  [68.48 - 98.35] | 78.84  [69.97 – 92.89] |
| Sample_4 (S4) | 203,359  [146,234 – 262,718] | 109,521  [79,721 – 139,239] | 96.98  [96.57 - 97.51] | 78.88  [73.75 – 87.27] |
| Sample_5 (S5) | 217,167  [147,614 – 287,748] | 117,283  [79,411 – 155,756] | 97.03  [96.30 - 97.99] | 78.83  [74.32 – 87.33] |
| Sample_6 (S6) | 230,620  [161,216 – 381,322] | 123,618  [84,962 – 206,942] | 97.06  [96.45 - 97.88] | 76.91  [71.53 – 86.69] |
| Sample_7 (S7) | 200,069  [51,750 – 311,278] | 107,571  [27,830 – 160,872] | 96.99  [91.35 - 98.18] | 75.51  [66.02 – 86.99] |
| Sample_8 (S8)^#^ | 316,744  [219,118 – 602,290] | 163,479  [115,353 – 302,674] | 92.69  [69.70 - 98.28] | 76.42  [66.05 – 95.94] |
| Sample_9 (S9) | 214,341  [160,794 – 294,724] | 115,007  [86,298 – 153,430] | 96.79  [93.65 - 97.78] | 75.70  [71.54 – 86.91] |
| Sample_10 (S10) | 203,282  [135,370 – 261,856] | 109,276  [73,007- 141,368] | 96.88  [96.32 - 97.67] | 74.61  [69.88 – 86.48] |
| Average^@^ | 269,057  (51,750 – 602,290) | 142,938  (27,830 – 302,674) | 96.12  (68.48 - 98.35) | 78.14  [66.02 – 95.94] |

The symbol (#) and (@) indicates authentic traditional medicines, and averaged across 160 Illumina MiSeq data sets. The symbol (*) indicates that the (pseudo)reads are the combined quality controlled (QC) pseudo-reads, and the QC processed unmerged forward and reverse reads.

**Table S8: Primer and probe sequence information of specific animal and plant species used in this study.**

| **Species** | **Targets** | **Primer/Probe** | **Sequence 5’-3’** | **Reference** |
| --- | --- | --- | --- | --- |
| *Zea mays* | *HMG* | ZM1-F | TTGGACTAGAAATCTCGTGCTGA | EU-RL-GMFF [1] |
|  |  | ZM1-R | GCTACATAGGGAGCCTTGTCCT |  |
|  |  | Probe ZM | 6-FAM-CAATCCACACAAACGCACGCGTA-TAMRA |  |
| *Glycine max* | *Le1* | Lec F | CCAGCTTCGCCGCTTCCTTC | EU-RL-GMFF [1] |
|  |  | Lec R | GAAGGCAAGCCCATCTGCAAGCC |  |
|  |  | Lec P | FAM-CTTCACCTTCTATGCCCCTGACAC-TAMRA |  |
| *Gossypium* sp*.* | *acp1* | acp1 primer1 | ATTGTGATGGGACTTGAGGAAGA | EU-RL-GMFF [1] |
|  |  | acp1 primer2 | CTTGAACAGTTGTGATGGATTGTG |  |
|  |  | acp1 probe | FAM-ATTGTCCTCTTCCACCGTGATTCCGAA-TAMRA |  |
| *Brassica napus* | *FatA* | FatA primer1 | GGTCTCTCAGCAAGTGGGTGAT | EU-RL-GMFF [1] |
|  |  | FatA primer2 | TCGTCCCGAACTTCATCTGTAA |  |
|  |  | FatA probe | FAM-ATGAACCAAGACACAAGGCGGCTTCA-TAMRA |  |
| *Triticum aestivum* | *Wx-1* | wx012-5' | GTCGCGGGAACAGAGGTGT | Iida et al. [2] |
|  |  | wx012-3' | GGTGTTCCTCCATTGCGAAA |  |
|  |  | wx012-T | FAM-CAAGGCGGCCGAAATAAGTTGCC-TAMRA |  |
| *Beta vulgaris* | *GS* | GluA3-F | GACCTCCATATTACTGAAAGGAAG | EU-RL-GMFF [1] |
|  |  | GluA3-R | GAGTAATTGCTCCATCCTGTTCA |  |
|  |  | GluD1 probe | FAM-CTACGAAGTTTAAAGTATGTGCCGCTC-TAMRA |  |
| *Meleagris gallopavo* | *melIL* | mellL-f | TGTATTTCAGTAGCACTGCTTATGACTACT | Laube et al. [3] |
|  |  | mellL-r | TTTATTAATGCTGGAAGAATTTCCAA |  |
|  |  | mellL-probe | 6-FAM-TTATGGAGCATCGCTATCACCAGAAAA-TAMRA |  |
| *Carica papaya* | CHY | Q-CHY-1F2 | CCATGCGATCCTCCCA | Prins et al. [4] |
|  |  | Q-CHY-2R | CATCGTAGCCATTGTAACACTAGCTAA |  |
|  |  | Q-CHY-P0 | FAM-TTCCCTTCAT-BHQ1-CCATTCCCACTCTTGAGA-P |  |
| *Solanum lycopersicum* | LAT52 | *Lat52-F* | AGACCACGAGAACGATATTTGC | Yang et al. [5] |
|  |  | *Lat52-R* | TTCTTGCCTTTTCATATCCAGACA |  |
|  |  | *Lat52-probe* | HEX-CTCTTTGCAGTCCTCCCTTGGGCT-BHQ |  |
| *Bos taurus* | BtaGH | BtaGH-1 | CCGATGGATGTGTTCAGAGCT | Brodmann et al. [6] |
|  |  | BtaGH-2 | GCCAAATGTCTGGGTGTAGATACC |  |
|  |  | BtaGH-S | 6-FAM-TGGGCTTTAGGGCTTCCGAATGTGAA-TAMRA |  |
| *Gallus gallus* | *galIL* | gallL-f | TGTTACCTGGGAGAAGTGGTTACT | Laube et al. [3] |
|  |  | gallL-r | TTTTCGATATTTTGAATAGCAGTTACAA |  |
|  |  | gallL-probe | 6-FAM-TGAAGAAAGAAACTGAAGATGACACTGAAATTAAAG-TAMRA |  |

**Table S9: qPCR regent composition for all the plant and animal targets used in this study.**

| **Component** | **Stock concentration** | **Final concentration** | **µl/reaction** |
| --- | --- | --- | --- |
| Diagenode 2x reaction buffer | 2x | 1x | 12.5 |
| Primer forward | 10 µM | 400 nM | 1.0 |
| Primer reverse | 10 µM | 400 nM | 1.0 |
| Probe | 10 µM | 200 nM | 0.5 |
| Water | – | – | 5.0 |
| DNA | 10 ng/µl | – | 5.0 |
| Total reaction volume | – | – | **25.0** |

**Table S10: qPCR program for all the plant and animal targets used in this study.**

| **Step** | | **T (^o^C)** | **Time (s)** | **Number of cycles** |
| --- | --- | --- | --- | --- |
| Decontamination UNG | | 50 | 120 | 1 |
| Denaturation | | 95 | 600 | 1 |
| Amplification | Denaturation | 95 | 15 | 45 |
|  | Annealing and extension | 60 | 60 |  |
| Hold |  | 20 | indefinitely | 1 |

**Reference:**

1. [<http://gmo-crl.jrc.ec.europa.eu/>]

2. Iida M, Yamashiro S, Yamakawa H, Hayakawa K, Kuribara H, Kodama T, Furui S, Akiyama H, Maitani T, Hino A: **Development of taxon-specific sequences of common wheat for the detection of genetically modified wheat.** *Journal of agricultural and food chemistry* 2005, **53:**6294-6300.

3. Laube I, Zagon J, Spiegelberg A, Butschke A, Kroh LW, Broll H: **Development and design of a ‘ready‐to‐use’reaction plate for a PCR‐based simultaneous detection of animal species used in foods.** *International journal of food science & technology* 2007, **42:**9-17.

4. Prins TW, Scholtens IM, Bak AW, van Dijk JP, Voorhuijzen MM, Laurensse EJ, Kok EJ: **A case study to determine the geographical origin of unknown GM papaya in routine food sample analysis, followed by identification of papaya events 16-0-1 and 18-2-4.** *Food Chemistry* 2016, **213:**536-544.

5. Yang L, Pan A, Jia J, Ding J, Chen J, Cheng H, Zhang C, Zhang D: **Validation of a tomato-specific gene, LAT52, used as an endogenous reference gene in qualitative and real-time quantitative PCR detection of transgenic tomatoes.** *Journal of agricultural and food chemistry* 2005, **53:**183-190.

6. Brodmann PD, Moor D: **Sensitive and semi-quantitative TaqMan™ real-time polymerase chain reaction systems for the detection of beef (Bos taurus) and the detection of the family Mammalia in food and feed.** *Meat Science* 2003, **65:**599-607.
