# Supplementary material for: Development and validation of a multi-locus DNA metabarcoding method to identify endangered species in complex samples
Source: Gigascience. 2017 Aug 19;6(10):1–18. doi: 10.1093/gigascience/gix080 (PMC5632295; doi:10.1093/gigascience/gix080)
Supplement: Response-to-Reviewer-Comments_Original-Submission.pdf [file gix080_Response-to-Reviewer-Comments_Original-Submission.pdf]

Author's Response To Reviewer Comments

Reviewer #1:

Reviewer #1: The manuscript entitled 'Development and validation of a multi-locus DNA metabarcoding method to identify endangered species in complex samples' provides a detailed analysis of 12 barcode markers applied to the identification of species within medicinal samples. This study also tests the validity and reproducibility of the developed metabarcoding method across 16 international laboratories. The authors have researched the topic area well, and have taken into account the most pertinent issues involved with attempting to identify species within degraded samples, and with making identifications using incomplete reference DNA databases. This is an invaluable study for the field of wildlife forensics, in particular with regard to endangered species identification in herbal medicines, and will hopefully help enforcement agencies towards prosecuting those involved in the illegal wildlife trade in the near future.

I have just a few questions for the authors in regards to some points in the methods.
1) How were the pooled libraries quantified (lines 642-643) prior to sequencing on the MiSeq? Could this information be added to this section?
The information on NGS library preparation was added to line 668-671: "The prepared Illumina libraries from each sample were quantified using the Quant-iT dsDNA broad range assay (Life Technologies). Furthermore, the normalised library pools were prepared and their concentration was quantified using KAPA library quantification kit (KAPA Biosystems) and pooled prior to MiSeq sequencing using MiSeq reagent kit v3."

2) Were all of the barcoding PCRs carried out using qPCR, and if so, were any of the DNA extracts deemed to be low copy number as evidenced by high CT values?
Thank you for raising this valuable question, however, no qPCRs were performed for the barcoding PCRs used in this study. The qPCR were performed only to check for potential cross-contamination using species-specific qPCRs during experimental sample preparation using the primers listed in Table S8 (additional File 1). This procedure was followed to confirm that indeed no cross-contamination had occurred, as explained on lines 410-412. No qPCRs were performed on the authentic TM samples.

3) Were extraction controls PCR amplified, and did any contain DNA? If so, were they sequenced as well? Could this be clarified in the methods please?
The extraction controls were checked with NanoDrop to see if they contained DNA. However, none of the samples of the extraction controls did or were reported to contain DNA. During PCR, a water control was used. Participants in the validation trial that observed PCR amplification in the negative control were asked to repeat the PCR reactions.
The following was added on DNA extraction: "….along with extraction control." (line 634), and "In case the extraction control contained DNA, the DNA isolation procedure was repeated" (line 637).
The following was added on PCR amplification: "….,together with positive and negative control reactions…" (line 653-654), and "If amplification was observed in the negative control, the PCR analysis was repeated." (line 654-655).

4) How did the authors choose the 46 reference samples included in the study, and why those species in particular?
The species were mainly selected to cover wide range of taxa (Mammalia, Actinopterygii, Malacostraca, Bivalvia, Aves, Reptilia, Amphibia, Insecta, Angiospermae, and Cycadopsida) which is specified in line 255 – 257 (and see Table S2 and S3, Additional file 1). CITES reference materials were received from Dutch Customs (line 587).

It is noted that the authors pooled 8uL of each PCR product to combine into a sample library. I just have a suggestion that in future metabarcoding library set-up, perhaps a method to quantify the concentration of the products could be carried out (e.g., fragment analyser if possible), and then library blending could be adjusted so that each amplicon is pooled in equimolar amounts. This could assist in gaining a more equal number of reads across each sample particularly where there are low read numbers of a genuine taxa that could otherwise be screened out in the bioinformatic filtering stages.

Thank you for this valuable suggestion for pooling equimolar amounts of amplicon from all the barcode markers, which might over the bias to obtain equal number of reads. However, in particular the problem is that Customs laboratories are not well equipped at this moment, they rely on simple and cheap techniques to provide the answers.

Reviewer #2: The authors presented us a mulilocus based metabarcoding study, in which several widespread biomarkers have been adopted to evaluate their efficacy on detection of endangered species and the efficacy has been validated in 15 wildlife forensics labs around the world. As we all know, taxonomic identification will be much more difficult in the case that only parts of an animal or plant without distinctive morphological characteristics are present, or they have been pulverized and have become ingredients of food or traditional medicines. Although metabarcoding has been introduced to estimate biodiversity from both mass samples and environmental DNAs for a long time, a standardized and high efficient method, in respect to safety and the trade of endangered species, to assess quality of complex mixture is in urgent need. However, there are several serious issues need to be addressed before it can be considered to be published.

Major:

1. According to the title, the authors aimed to detect CITES list species, however, the analysis pipeline hasn't showed any CITES tailored specifics. if it has, the authors may want to emphasize them out. The pipeline is, after all, named after CITESspeciesDetect. In addition, the authors may want to build their own biomarker reference for species in CITES list and modify their taxonomic identification methods, for example, several taxonomic assignment programs have been developed, such as Probabilistic method for taxonomical classification (PROTAX) [Paun et al. Bioinformatics (2016) 32 (19): 2920-2927], methods related to multi-locus species clustering [Douglas et al., Methods in Ecology and Evolution 2013, 4, 961-970].

The pipeline is modified to accommodate CITES species identification in such a way that the BLAST output is automatically matched to the speciesplus.net database. The taxonomic level at which the identified species is listed by speciesplus.net is automatically added to the BLAST output table, together with the CITES appendix (I, II or III) at which it is listed. Speciesplus.net contains up-to-date and official information on CITES listings, and is a collaboration between UNEP-WCMC and CITES (www.speciesplus.net). Details describing this aspect of the pipeline can be found in line 705-716, and figure 1. Also, the web-interface facilitates (even more) intuitive BLAST identification of species listed by speciesplus.net by highlighting species on CITES appendix I in red. Species listed on CITES appendix II and II are highlighted in orange and yellow, respectively.
We realise that the above feature of the web-interface was not described in the text. Therefore, the following lines were added: “The web-interface facilitates intuitive BLAST identification of species listed by speciesplus.net by highlighting species on CITES appendix I in red. Species listed on CITES appendix II and II are highlighted in orange and yellow, respectively.” (lines 726– 728).
It is a valuable suggestion to create a CITES species specific biomarkers database for identification. However, as discussed (lines 551 – 560), CITES species are highly underrepresented in current sequence databases (including BOLD). Also, reliable identification of CITES species requires a good coverage of species related to CITES taxa in sequence databases. This, together with the wide diversity of endangered plant and animal species (>35000 species listed by CITES) restricted us to use the NCBI nt database. Certainly, initiatives that may help to improve coverage of CITES taxa in sequence databases such as Barcode of Wildlife are needed, allowing us to update the BLAST database of the CITESspeciesDetect pipeline, but this fall out of the scope of this study.
We do not have hands-on experience with the PROTAX identification method and other mentioned methods, but they seem very interesting. We decided to use the BLAST identification method because it is very well established and has proven to work well in many DNA (meta)barcoding studies e.g. Fahner et al. 2016 http://journals.plos.org/plosone/article?id=10.1371/journal.pone.0157505; van Velzen et al. (2012; http://journals.plos.org/plosone/article?id=10.1371/journal.pone.0030490. BOLD also makes use of BLAST for plant species identification (http://v4.boldsystems.org/). Nonetheless, we are very much interested in keeping up-to-date with new methods, and we are keen on incorporating them once they have proven to be valuable for routine identification.

2. It states that "The method provides improved resolution for species identification, while verifying species with multiple DNA barcodes contributes to an enhanced quality assurance" at line 108-109, and reiterate it in several other places. However, according to current pipeline, multiple biomarkers adopted here may only enhance sensitivity, rather than quality. Do current pipeline require at least 2 biomarkers, for example, to verify the present of targeted species?
There is no criterion of the pipeline that at least 2 biomarkers are needed to confirm the presence of a species. What we advocate is that the use of multiple biomarkers can improve quality assurance i.e. confirming species with multiple barcodes is perceived by the end-user with higher confidence than identifications based on a single barcode marker (see also tables 4 and 5).
In order to improve the readability on this point, we modified the following sentence: “The method can provide improved resolution for species identification, while verifying species with multiple DNA barcodes contributes to an enhanced quality assurance” (line 106). Line 493-495

was removed, because it may have implied that all species were identified using multiple DNA barcodes.

3a. The authors claimed that both cytb and COI cannot be separated with their corresponding mini-barcodes at Line 281-285. Firstly, pair end reads contain both 5' primer and 3' primer info, which makes the separation feasible. In addition, 300 PE reads can read through mini-barcode, however, not their corresponding long ones, so it won't be a hard job to separate each other.
The reviewer is correct that it is certainly technically possible to separate full-length COI, and mini-COI barcodes (and full-length cyt b, and mini cyt b barcodes). We agree that line 282-284 implies that this is not technically possible, which is indeed incorrect. Therefore, line 282-284 was modified into: "It was found that with the current setup of the pipeline, reads generated for cyt b and mini-cyt b could not be separated based on the forward PCR primer, as the forward primers are identical." Also, we added to the discussion (line 499-502) a sentence with modifications to improve the pipeline in future work. "The design of the pipeline prevented cyt b and COI full length barcodes to be separated from their corresponding mini-barcodes, as they have identical forward primers. Since, the 300 PE reads can read through the cyt b and COI mini-barcodes, and therefore contain both 5'primer and 3'primer information, separation should be feasible."

It also relates to my concerns with regard to your current analysis pipeline in several other aspects:
1) PREINSEQ is supposed to be adopted at the very first step so as to remove low quality reads;
Our data sets consist of part reads that can be merged (small barcodes <~550 bp) and reads that cannot be merged (>~550bp). Quality filtering of reads before merging will prevent reads from overlapping in many cases, altogether preventing them from merging. We have following the instructions of the USEARCH manual on this: http://drive5.com/usearch/manual/pipe_readprep_merge.html. Quality trimming is performed after merging for mergeable reads as recommended by http://drive5.com/usearch/manual/ex_min2.html
Reads that cannot be merged are basically first quality trimmed using PrinSeq, as indicated on line 687-688-. This was indeed done to prevent many low quality reads from passing into the clustering steps.

2) Line 297-301, adapter removal and barcode assignment should be 2 different steps and shouldn't be analyzed and discussed at the same time. In addition, allowing no mismatch will inevitably leave some reads with unremoved adapter, which is obvious and unnecessary to be shown here;
Cutadapt does primer selecting, trimming and sorting in a single step (line 690). We therefore included this in the same paragraph. We agree that the effects of some of the tested parameters are predictable and, perhaps, common-knowledge. We therefore decided to remove the paragraph on Illumina adapter trimming altogether (line 298-302).

3) Line 309-314, when the authors separated all your barcodes to corresponding catalogues, their length distribution parameter should of course be set to various length, as the authors have

already summarized in table 1, the length distribution of different biomarkers varies a lot.
We indeed applied variable length filtering setting for different barcodes. For ITS2 a 100 nt minimum threshold was applied, for rbcL-mini this was 140 nt, and trnL-P6loop we used 10 nt. All, other barcodes (merged or unmerged) had length cut-offs of >200nt, as can be seen in https://github.com/RIKILT/CITESspeciesDetect/blob/master/CheckCriteriaBlastSingleSample.py. We realise this was not clear from the text and therefore updated line 315-318: “We implemented a minimum DNA barcode length of 200 nt, except for DNA barcodes with a basic length shorter than 200 nt, in which case the minimum expected DNA barcode length is set to 100 nt for ITS2, 140 nt for mini-rbcL, and 10 nt for the trnL (P6 loop) marker.”

4. Data volume will affect species present/absence a lot, especially on species of low abundant. Therefore, what the threshold set in this study can be invalidate or inappropriate for different data set. Since it contained several cross-lab validations and different labs generated various data volume, which can be used to estimate the effect of data volume on parameter adjustment. For example, at line 672-673, minimum cluster size of 4 is set, readers would be interested in the effect of data volume on the threshold and as far as I know, quite a lot studies removed singleton reads only.

We agree with the reviewer's reasoning that the threshold should depend on the data volume. Here, the minimum cluster size was set to reduce the number of spurious clusters containing singletons and bad sequences. The default setting is 2 (http://drive5.com/usearch/manual/singletons.html), but higher settings are not uncommon. For instance, Farner et al (2016: http://journals.plos.org/plosone/article?id=10.1371/journal.pone.0157505) used a setting of 10. The setting of 4 is extremely low considering the rather low diversity of plant and animal species expected in typical forensic samples vs. the high sequencing depth used in this study (Table 7-9, Additional file 1 Table S7). Moreover, the minimum cluster size is in all cases overruled by the 0.2% OTU abundance, which is an additional filter on top of minimum cluster size filtering, and which can be considered as the real abundance threshold. Indeed the latter OTU abundance threshold is scaled to data volume.

5. I agree that a lower limit threshold of OUT abundance (0.2% in this study) should be set, however, the authors may want to clarity that this will lead to false negative for ingredients which is relatively low abundant in the mixture, for example, only account for < 0.2% dry weight in the mixture.

We agree with your concern that an OTU abundance threshold of 0.2% might be incorrect to apply for identifying species present at < 1%. Therefore, a statement was added on line 308 – 311: “It should be noted that applying filtering thresholds may always lead to false negative or false positive identifications. In this study, an OTU abundance threshold of 0.2% was set as default, however, the OTU abundance threshold may need re-evaluation for samples with expected very low species abundances (<1% dry weight).”

6. The authors should be much more careful when submitted their manuscripts. The tables should be well organized, for instance, columns after EM 11 in table 2 cannot be displayed.

We agree that the tables do not fully fit in the page layout, that's why we submitted the tables in a separate word document, so it can be viewed via web layout option and we also thought the journal will format the tables. Now, we modified the table 2 slightly so most of the tables can fit in the page layout.

7. Mito-genomics (Tang et al. Method in Ecology and Evolution. 2015. 6, 1034-1043) combined with capture tech (Liu et al. Molecular Ecology Resource. 2016. 16(2), 470-479) can also be a promising methodology, which can tackle issues of highly degraded samples and lack of universal PCR primer since it circumvents PCR step. The authors may want to add this in the introduction or discussion part.

This suggestion by the reviewer is undeniable, we took the opportunity to incorporate this suggestion in the discussion section (line 548-550) and added the specified reference to the text in the manuscript (reference 23 and 25): "A PCR-free targeted DNA capturing approach coupled with shotgun sequencing was recently proposed for biodiversity assessments which may potentially also be suitable for enhancing species identification in difficult wildlife forensic samples [23, 25]."

Minor:

Line 140: "would not possible" is NOT true. Sanger sequencing can accomplish such job but being more complicated, for example using clone picking.

Based on you remark, we now changed this into (line 141): "….would not be possible through morphological means and time-consuming with traditional DNA barcoding."

Line 207-210, Bioinformatics procedures should not be included in the data description part.

We removed the bioinformatics procedures from the data description (line 208-211).

Line 244, please unify the symbol of COI biomarker.

Thank you for spotting out, we unify the COI abbreviation through the manuscript and additional files.

Contamination issues at line 358-367, do these putative contamination infer from multiple biomarkers? It needs more details. BTW, include negative control sample in both DNA extraction and PCR step would be a good idea for contamination issues, the authors may want to add this in the discussion and do such in their further work.

This is a good point indeed, the cross-contamination was observed from multiple biomarkers, a detail information about the cross-contaminated species and related marker can be found in the additional file 2; Table S1.
We also added additional text that the Cq value of the qPCR was above 39, which indicated the

presence of the species, however, in low copy number. Reviewer 1 also had a similar comment about the use of a negative control, we acknowledge that this is not clearly presented in the manuscript, taking the opportunity to clarify, we used a negative control in both DNA isolation and PCR reaction, which is now clarified in the manuscript under the methods section. The following was added on PCR amplification: "….,together with positive and negative control reactions…" (line 653-654), and "If amplification was observed in the negative control, the PCR analysis was repeated." (line 654-655).

Line 371-373, do these removed biomarkers have any trends? Are they tend to be the same biomarkers? Similar length? Or various without any particular traits?

We had another look into the data to find why these species (Parapenaeopsis sp., A. anguilla, and C. revoluta) are not identified and we found no trend. We therefore added the following to the text (line 380-382) "There appeared to be no trend as to the type and length of DNA barcode marker that had been filtered out by the CITESspeciesDetect pipeline. For instance, Parapenaeopsis sp. was detected below the OTU threshold with cyt b, mini-16S, COI, and 16S markers (not shown)."

Line 478-480, if authors include this point here as one of its major conclusions, it is better to include the comparison results in this study, may be in the supplementary file at least.

No thorough comparison of DNA isolation methods has been made in this study. Based on our previous in-house experiences with plant and animal reference samples, GMO feed materials, and other processed real-life samples we decide to go for the CTAB DNA isolation method. We therefore included recommendations for future work (line 486-491): "As a first step, a CTAB DNA isolation method was selected for efficiently extracting high quality DNA from pure plant and animal reference materials as well as from complex mixtures. DNA isolation can be very difficult to standardise and optimise because of the complexity and diversity of wild life forensic samples, and a more systematic comparison of different DNA extraction methods is required."

Line 500 - 502, please make it clear.

To improve readability we changed the structure of the sentence (line 515-518): "However, not all laboratories could identify all specified ingredients (species) in the analysed experimental samples. From the current study, we demonstrate that diverse animal taxa could be identified at the species level, which highlights the objective of the method to target a wide range of animal species.".

Line 517-518, it can also be resolved by efforts on bioinformatics, e g. [Liu et al. Method in Ecology and Evolution, 2013, 4(12), 1142-1150]

Thanks for the suggestion. We added to article to line (534-536): "Alternatively, full-length barcodes may be resolved using an advanced bioinformatics strategy (SOAPBarcode) to assemble Illumina shotgun sequences of PCR amplicons".

Line 534-535, please give a rough estimation of this underrepresentation.

We have estimated the number of CITES-listed species with DNA barcodes (COI for animals, and matK or rbcL for plants) using edirect and the 35787 species on the CITES list to query the NCBI nucleotide database. We found that there are only 6742 species with at least one barcode marker, representing 18.8% of total.
On line 555-557 we included: “We estimate that only 18.8% of species on the CITES list contain one or more DNA barcodes (COI for animals, and matK or rbcL for plants).”
