# Supplementary material for: Development and validation of a multi-locus DNA metabarcoding method to identify endangered species in complex samples
Source: Gigascience. 2017 Aug 19;6(10):1–18. doi: 10.1093/gigascience/gix080 (PMC5632295; doi:10.1093/gigascience/gix080)
Supplement: Response-to-Reviewer-Comments_Revision-1.pdf [file gix080_Response-to-Reviewer-Comments_Revision-1.pdf]

Author's Response To Reviewer Comments

Reviewer reports:
Reviewer #2: Most of the concerns have been well addressed except for major issue #4 - validation across different labs generated disparate data volume for same samples, which is supposed to be valuable resource to study the effect of sequencing data output size on parameter selection, especially on those cutoffs, e. g. minimum cluster size. Please include a part talking about such findings. And the paper can be accepted after this adding.

We have performed additional analyses to provide insight on the effect of two important parameters (OTU minimum cluster size and OTU abundance threshold) on identifying the low abundant species Beta vulgaris in three samples with low, medium and high read counts.
We have included the following paragraph (line 439 – 448):
"In order to provide insight into what alternative setting of the CITESspeciesDetect pipeline may have been better suited for identifying Beta vulgaris, three data sets with relatively low (S6 – laboratory 13), medium (S6 – laboratory 14) and high (S6 – laboratory 6) data volumes were reanalysed using a range of different settings for the OTU minimum cluster size and OTU abundance threshold (Additional file 2: Table S3-S5). Setting the OTU minimum cluster size to 2, 4, or 6 has no effect on taxon identification, and Beta vulgaris is not identified at the species or higher taxonomic level in the data sets of laboratories 6 and 13. Setting the OTU abundance threshold to zero allows identifying Beta vulgaris in all three samples, but at the expense of many false positive identifications. Applying an OTU abundance threshold of 0.1% (default is 0.2%) allows identifying Beta vulgaris at the species or genus level irrespective of any differences in data volume between the three samples.

Also, we included a table with the exact read counts for Beta vulgaris in all 16 data sets for sample 6.
For this we included the additional table S2 in additional file 2 and updated in the manuscript line 438

We also updated the Additional files session:
The newly added tables title were added to the manuscript : "line 799-803"
SOP developed in this study was uploaded to the protocols.io and DOI was obtained, so the DOI was used in the manuscript to refer the SOP in "line 388" and "line 746"

Additional file 3: Standard operating procedure (SOP) for the multi-locus DNA metabarcoding method that was used in the inter-laboratory validation study (*.pdf) was removed from the manuscript " line 806-807"
Additional file 4 was changed to Additional file 3 and updated in the manuscript: "line 214" "line 810"
