# Supplementary material for: Development and validation of a multi-locus DNA metabarcoding method to identify endangered species in complex samples
Source: Gigascience. 2017 Aug 19;6(10):1–18. doi: 10.1093/gigascience/gix080 (PMC5632295; doi:10.1093/gigascience/gix080)
Supplement: Reviewer-1-Report-(Original-Submission).pdf [file gix080_Reviewer-1-Report-(Original-Submission).pdf]

Reviewer Report

Title: Development and validation of a multi-locus DNA metabarcoding method to identify endangered species in complex samples

Version: Original Submission **Date:** 5/23/2017

Reviewer name: Megan Coghlan

Reviewer Comments to Author:

The manuscript entitled 'Development and validation of a multi-locus DNA metabarcoding method to identify endangered species in complex samples' provides a detailed analysis of 12 barcode markers applied to the identification of species within medicinal samples. This study also tests the validity and reproducibility of the developed metabarcoding method across 16 international laboratories. The authors have researched the topic area well, and have taken into account the most pertinent issues involved with attempting to identify species within degraded samples, and with making identifications using incomplete reference DNA databases. This is an invaluable study for the field of wildlife forensics, in particular with regard to endangered species identification in herbal medicines, and will hopefully help enforcement agencies towards prosecuting those involved in the illegal wildlife trade in the near future.

I have just a few questions for the authors in regards to some points in the methods.
1) How were the pooled libraries quantified (lines 642-643) prior to sequencing on the MiSeq? Could this information be added to this section?
2) Were all of the barcoding PCRs carried out using qPCR, and if so, were any of the DNA extracts deemed to be low copy number as evidenced by high CT values?
3) Were extraction controls PCR amplified, and did any contain DNA? If so, were they sequenced as well? Could this be clarified in the methods please?
4) How did the authors choose the 46 reference samples included in the study, and why those species in particular?

It is noted that the authors pooled 8uL of each PCR product to combine into a sample library. I just have a suggestion that in future metabarcoding library set-up, perhaps a method to quantify the concentration of the products could be carried out (e.g., fragment analyser if possible), and then library blending could be adjusted so that each amplicon is pooled in equimolar amounts. This could assist in gaining a more equal number of reads across each sample particularly where there are low read numbers of a genuine taxa that could otherwise be screened out in the bioinformatic filtering stages.

Methods

Are the methods appropriate to the aims of the study, are they well described, and are necessary controls included? Yes

Conclusions

Are the conclusions adequately supported by the data shown? Yes

Reporting Standards

Does the manuscript adhere to the journal's guidelines on minimum standards of reporting? YesChoose an item.

Statistics

Are you able to assess all statistics in the manuscript, including the appropriateness of statistical tests used? There are no statistics in the manuscript.

Quality of Written English

Please indicate the quality of language in the manuscript: Acceptable

Declaration of Competing Interests

Please complete a declaration of competing interests, considering the following questions:

- Have you in the past five years received reimbursements, fees, funding, or salary from an organisation that may in any way gain or lose financially from the publication of this manuscript, either now or in the future?
- Do you hold any stocks or shares in an organisation that may in any way gain or lose financially from the publication of this manuscript, either now or in the future?
- Do you hold or are you currently applying for any patents relating to the content of the manuscript?
- Have you received reimbursements, fees, funding, or salary from an organization that holds or has applied for patents relating to the content of the manuscript?
- Do you have any other financial competing interests?
- Do you have any non-financial competing interests in relation to this paper?

If you can answer no to all of the above, write 'I declare that I have no competing interests' below. If your reply is yes to any, please give details below.

I declare that I have no competing interests

I agree to the open peer review policy of the journal. I understand that my name will be included on my report to the authors and, if the manuscript is accepted for publication, my named report including any attachments I upload will be posted on the website along with the authors' responses. I agree for my report to be made available under an Open Access Creative Commons CC-BY license

(http://creativecommons.org/licenses/by/4.0/). I understand that any comments which I do not wish to be included in my named report can be included as confidential comments to the editors, which will not be published.

I agree to the open peer review policy of the journal

To further support our reviewers, we have joined with Publons, where you can gain additional credit to further highlight your hard work (see: https://publons.com/journal/530/gigascience). On publication of this paper, your review will be automatically added to Publons, you can then choose whether or not to claim your Publons credit. I understand this statement.

Yes
