# Supplementary material for: Development and validation of a multi-locus DNA metabarcoding method to identify endangered species in complex samples
Source: Gigascience. 2017 Aug 19;6(10):1–18. doi: 10.1093/gigascience/gix080 (PMC5632295; doi:10.1093/gigascience/gix080)
Supplement: Reviewer-2-Report-(Original-Submission).pdf [file gix080_Reviewer-2-Report-(Original-Submission).pdf]

Reviewer Report

Title: Development and validation of a multi-locus DNA metabarcoding method to identify endangered species in complex samples

Version: Original Submission **Date:** 5/25/2017

Reviewer name: Shanlin Liu

Reviewer Comments to Author:

The authors presented us a mulilocus based metabarcoding study, in which several widespread biomarkers have been adopted to evaluate their efficacy on detection of endangered species and the efficacy has been validated in 15 wildlife forensics labs around the world. As we all know, taxonomic identification will be much more difficult in the case that only parts of an animal or plant without distinctive morphological characteristics are present, or they have been pulverized and have become ingredients of food or traditional medicines. Although metabarcoding has been introduced to estimate biodiversity from both mass samples and environmental DNAs for a long time, a standardized and high efficient method, in respect to safety and the trade of endangered species, to assess quality of complex mixture is in urgent need. However, there are several serious issues need to be addressed before it can be considered to be published.

Major:

1. According to the title, the authors aimed to detect CITES list species, however, the analysis pipeline hasn't showed any CITES tailored specifics. if it has, the authors may want to emphasize them out. The pipeline is, after all, named after CITESspeciesDetect. In addition, the authors may want to build their own biomarker reference for species in CITES list and modify their taxonomic identification methods, for example, several taxonomic assignment programs have been developed, such as Probabilistic method for taxonomical classification (PROTAX) [Paun et al. Bioinformatics (2016) 32 (19): 2920-2927], methods related to multi-locus species clustering [Douglas et al., Methods in Ecology and Evolution 2013, 4, 961-970].

2. It states that "The method provides improved resolution for species identification, while verifying species with multiple DNA barcodes contributes to an enhanced quality assurance" at line 108-109, and reiterate it in several other places. However, according to current pipeline, multiple biomarkers adopted here may only enhance sensitivity, rather than quality. Do current pipeline require at least 2 biomarkers, for example, to verify the present of targeted species?

3. The authors claimed that both cytb and COI cannot be separated with their corresponding mini-barcodes at Line 281-285. Firstly, pair end reads contain both 5' primer and 3' primer info, which makes

the separation feasible. In addition, 300 PE reads can read through mini-barcode, however, not their corresponding long ones, so it won't be a hard job to separate each other. It also relates to my concerns with regard to your current analysis pipeline in several other aspects: 1) PREINSEQ is supposed to be adopted at the very first step so as to remove low quality reads; 2) Line 297-301, adapter removal and barcode assignment should be 2 different steps and shouldn't be analyzed and discussed at the same time. In addition, allowing no mismatch will inevitably leave some reads with unremoved adapter, which is obvious and unnecessary to be shown here; 3) Line 309-314, when the authors separated all your barcodes to corresponding catalogues, their length distribution parameter should of course be set to various length, as the authors have already summarized in table 1, the length distribution of different biomarkers varies a lot.

4. Data volume will affect species present/absence a lot, especially on species of low abundant. Therefore, what the threshold set in this study can be invalidate or inappropriate for different data set. Since it contained several cross-lab validations and different labs generated various data volume, which can be used to estimate the effect of data volume on parameter adjustment. For example, at line 672-673, minimum cluster size of 4 is set, readers would be interested in the effect of data volume on the threshold and as far as I know, quite a lot studies removed singleton reads only.

5. I agree that a lower limit threshold of OUT abundance (0.2% in this study) should be set, however, the authors may want to clarity that this will lead to false negative for ingredients which is relatively low abundant in the mixture, for example, only account for < 0.2% dry weight in the mixture.

6. The authors should be much more careful when submitted their manuscripts. The tables should be well organized, for instance, columns after EM 11 in table 2 cannot be displayed.

7. Mito-genomics (Tang et al. Method in Ecology and Evolution. 2015. 6, 1034-1043) combined with capture tech (Liu et al. Molecular Ecology Resource. 2016. 16(2), 470-479) can also be a promising methodology, which can tackle issues of highly degraded samples and lack of universal PCR primer since it circumvents PCR step. The authors may want to add this in the introduction or discussion part.

Minor:

Line 140: "would not possible" is NOT true. Sanger sequencing can accomplish such job but being more complicated, for example using clone picking.

Line 207-210, Bioinformatics procedures should not be included in the data description part.

Line 244, please unify the symbol of COI biomarker.

Contamination issues at line 358-367, do these putative contamination infer from multiple biomarkers? It needs more details. BTW, include negative control sample in both DNA extraction and PCR step would be a good idea for contamination issues, the authors may want to add this in the discussion and do such in their further work.

Line 371-373, do these removed biomarkers have any trends? Are they tend to be the same biomarkers? Similar length? Or various without any particular traits?

Line 478-480, if authors include this point here as one of its major conclusions, it is better to include the comparison results in this study, may be in the supplementary file at least.

Line 500 - 502, please make it clear.

Line 517-518, it can also be resolved by efforts on bioinformatics, e g. [Liu et al. Method in Ecology and Evolution, 2013, 4(12), 1142-1150]

Line 534-535, please give a rough estimation of this underrepresentation.

Methods

Are the methods appropriate to the aims of the study, are they well described, and are necessary controls included? Yes

Conclusions

Are the conclusions adequately supported by the data shown? Yes

Reporting Standards

Does the manuscript adhere to the journal's guidelines on minimum standards of reporting? YesChoose an item.

Statistics

Are you able to assess all statistics in the manuscript, including the appropriateness of statistical tests used? Yes, and I have assessed the statistics in my report.

Quality of Written English

Please indicate the quality of language in the manuscript: Acceptable

Declaration of Competing Interests

Please complete a declaration of competing interests, considering the following questions:

- Have you in the past five years received reimbursements, fees, funding, or salary from an organisation that may in any way gain or lose financially from the publication of this manuscript, either now or in the future?
- Do you hold any stocks or shares in an organisation that may in any way gain or lose financially from the publication of this manuscript, either now or in the future?
- Do you hold or are you currently applying for any patents relating to the content of the manuscript?
- Have you received reimbursements, fees, funding, or salary from an organization that holds or has applied for patents relating to the content of the manuscript?
- Do you have any other financial competing interests?
- Do you have any non-financial competing interests in relation to this paper?

If you can answer no to all of the above, write 'I declare that I have no competing interests' below. If your reply is yes to any, please give details below.

I declare that i have no competing interests

I agree to the open peer review policy of the journal. I understand that my name will be included on my report to the authors and, if the manuscript is accepted for publication, my named report including any attachments I upload will be posted on the website along with the authors' responses. I agree for my report to be made available under an Open Access Creative Commons CC-BY license (http://creativecommons.org/licenses/by/4.0/). I understand that any comments which I do not wish to be included in my named report can be included as confidential comments to the editors, which will not be published.

I agree to the open peer review policy of the journal

To further support our reviewers, we have joined with Publons, where you can gain additional credit to further highlight your hard work (see: https://publons.com/journal/530/gigascience). On publication of this paper, your review will be automatically added to Publons, you can then choose whether or not to claim your Publons credit. I understand this statement.

Yes
